# CERT_L_ reduces C16 ceramide, amyloid-β levels, and inflammation in a model of Alzheimer’s disease

**DOI:** 10.1186/s13195-021-00780-0

**Published:** 2021-02-17

**Authors:** Simone M. Crivelli, Qian Luo, Jo A.A. Stevens, Caterina Giovagnoni, Daan van Kruining, Gerard Bode, Sandra den Hoedt, Barbara Hobo, Anna-Lena Scheithauer, Jochen Walter, Monique T. Mulder, Christopher Exley, Matthew Mold, Michelle M. Mielke, Helga E. De Vries, Kristiaan Wouters, Daniel L. A. van den Hove, Dusan Berkes, María Dolores Ledesma, Joost Verhaagen, Mario Losen, Erhard Bieberich, Pilar Martinez-Martinez

**Affiliations:** 1 Department of Psychiatry and Neuropsychology, School for Mental Health and Neuroscience, Maastricht University, Universiteitssingel 50, 6229 ER Maastricht, the Netherlands; 2 Department of Physiology, University of Kentucky College of Medicine, Lexington, KY USA; 3 Veterans Affairs Medical Center, Lexington, KY 40502 USA; 4 Department of Internal Medicine, Laboratory Vascular Medicine, Erasmus MC University Medical Center, Rotterdam, the Netherlands; 5 Laboratory for Neuroregeneration, Netherlands institute for Neuroscience, Amsterdam, the Netherlands; 6 Department of Neurology, University Hospital Bonn, University of Bonn, Bonn, Germany; 7 The Birchall Centre, Lennard-Jones Laboratories, Keele University, Staffordshire, UK; 8 Division of Epidemiology, Department of Health Science Research, and Department of Neurology, Mayo Clinic Rochester, Rochester, MN USA; 9 Department of Molecular Cell Biology and Immunology, Amsterdam Neuroscience, Amsterdam UMC, Amsterdam, the Netherlands; 10 Department of Internal Medicine, Maastricht University Medical Centre, Maastricht, the Netherlands; 11 Cardiovascular Research Institute Maastricht (CARIM), Maastricht, the Netherlands; 12 Department of Psychiatry, Psychosomatics and Psychotherapy, University of Wuerzburg, Wuerzburg, Germany; 13 Department of Organic Chemistry, Slovak University of Technology, Radlinského 9, 81237 Bratislava, Slovak Republic; 14 Department of Molecular Neuropathology, Centro de Biología Molecular “Severo Ochoa” (CSIC-UAM), Madrid, Spain

**Keywords:** Ceramide, Sphingomyelin, Ceramide transporter protein (CERT), Adeno-associated virus (AAV), Alzheimer’s disease (AD), 5xFAD, Amyloid-β plaques, Neuroinflammation, Microglia

## Abstract

**Background:**

Dysregulation of ceramide and sphingomyelin levels have been suggested to contribute to the pathogenesis of Alzheimer’s disease (AD). Ceramide transfer proteins (CERTs) are ceramide carriers which are crucial for ceramide and sphingomyelin balance in cells. Extracellular forms of CERTs co-localize with amyloid-β (Aβ) plaques in AD brains. To date, the significance of these observations for the pathophysiology of AD remains uncertain.

**Methods:**

A plasmid expressing CERT_L_, the long isoform of CERTs, was used to study the interaction of CERT_L_ with amyloid precursor protein (APP) by co-immunoprecipitation and immunofluorescence in HEK cells. The recombinant CERT_L_ protein was employed to study interaction of CERT_L_ with amyloid-β (Aβ), Aβ aggregation process in presence of CERT_L_, and the resulting changes in Aβ toxicity in neuroblastoma cells. CERT_L_ was overexpressed in neurons by adeno-associated virus (AAV) in a mouse model of familial AD (5xFAD). Ten weeks after transduction, animals were challenged with behavior tests for memory, anxiety, and locomotion. At week 12, brains were investigated for sphingolipid levels by mass spectrometry, plaques, and neuroinflammation by immunohistochemistry, gene expression, and/or immunoassay.

**Results:**

Here, we report that CERT_L_ binds to APP, modifies Aβ aggregation, and reduces Aβ neurotoxicity in vitro. Furthermore, we show that intracortical injection of AAV, mediating the expression of CERT_L_, decreases levels of ceramide d18:1/16:0 and increases sphingomyelin levels in the brain of male 5xFAD mice. CERT_L_ in vivo over-expression has a mild effect on animal locomotion, decreases Aβ formation, and modulates microglia by decreasing their pro-inflammatory phenotype.

**Conclusion:**

Our results demonstrate a crucial role of CERT_L_ in regulating ceramide levels in the brain, in amyloid plaque formation and neuroinflammation, thereby opening research avenues for therapeutic targets of AD and other neurodegenerative diseases.

**Supplementary Information:**

The online version contains supplementary material available at 10.1186/s13195-021-00780-0.

## Background

Key pathological features of Alzheimer’s disease (AD) are aggregates of amyloid-β peptides (Aβ) and neurofibrillary tangles (NFTs), and neurodegeneration, together with blood-brain barrier (BBB) dysfunction, neuroinflammation, and lipid disbalance. To date, the molecular mechanism underlying neurodegeneration in AD remains unclear. Elucidation of the dysregulated biological mechanisms that lead to the onset and progression of AD is critical to identify new treatment strategies [1–5].

Sphingolipids (SLs) are waxy lipids formed by a sphingosine backbone, important for the cell membrane architecture and for the function of transmembrane proteins. Furthermore, SLs such as ceramides (Cer) and sphingosine-1-phosphate (S1P) are potent second messengers that regulate various important cellular processes, including cell growth and apoptosis [6–9]. Cer are formed by two metabolic pathways: de novo synthesis initiated with the precursor palmitoyl-CoA, or catabolism of complex SLs such as sphingomyelin (SM) [10–12]. In the cell membrane, SLs are typically organized in microdomains, called lipid rafts, characterized by specific SL species composition.

Several studies have analyzed lipid composition in AD brain tissue, reporting an increase of Cer species [13–16]. Lipid rafts enriched in Cer, isolated postmortem from frontal cortex tissue of AD patients, showed a reduction of SM levels compared to those isolated from the control brains [17]. Moreover, in AD brain tissue, SM levels were reduced in brain regions particularly vulnerable to Aβ plaque formation [18–20]. Strong evidence links Aβ pathology to SL homeostasis. The enzymes β-secretase and γ-secretase, which cleave the amyloid precursor protein (APP) to generate Aβ, are stabilized and have an increased half-life in Cer enriched membranes, thus increasing Aβ biogenesis [21, 22]. In turn, Aβ can stimulate Cer production by directly activating the phosphodiesterase enzyme sphingomyelinase which converts SM to Cer [18, 19].

Ceramide transfer proteins (CERTs) contain a steroidogenic acute regulatory protein (StAR)-related lipid transfer (START) domain that confers the ability to transport Cer intracellularly between the endoplasmic reticulum (ER) and the Golgi [23]. CERTs are found in at least two isoforms, which differ for the presence of a 26-amino acid serine-rich domain [24]. CERTs are expressed in the central nervous system, and they are crucial in embryogenesis and brain development [24–26]. When CERTs’ activity is blocked pharmacologically or genetically, by compromising the START domain of the protein, SM production decreases significantly [27, 28]. CERT_L_ can be secreted extracellularly and was found to partially co-localize with serum amyloid P component (SAP) and with amyloid plaques in AD brain [29, 30]. Besides, CERTs are also potent activators of the classical complement pathway that plays an active role in AD pathogenesis [31]. To date, the significance of these observations for the pathophysiology of AD remains uncertain.

In the current study, we investigated the interaction of CERT_L_ with APP and Aβ in vitro. Next, we explored the effect of CERT_L_ overexpression on SL composition, amyloid formation, and inflammation in vivo using adeno-associated virus (AAV)-mediated gene delivery in the 5xFAD mouse model [32]. Our findings showed that an increase of CERT_L_ modulated SL levels by reducing specific Cer and elevating SM. Notably, CERT_L_ also affected amyloid plaque formation and brain inflammation, supporting the idea that enzymes and transporters of the SL pathways are at the core of the pathophysiological changes observed in AD.

## Material and methods

### CERT_L_ interaction with APP/Aβ

#### Immunoprecipitation (IP)

Wild-type HEK293 and transgenic HEK293 cells that stably overexpress human APP695 isoform (NP_958817.1) [33] were cultured in Dulbecco’s modified Eagle’s medium (DMEM) supplemented with fetal bovine serum (FBS), penicillin/streptomycin (Pen/Strep), and L-glutamine. Stable transfected HEK-APP were maintained in G418 selective medium. Prior to the experiment, cells were seeded in 25-cm^2^ flasks and maintained in serum-free DMEM for 24 h. For the homogenization, cells were washed two times with phosphate-buffered saline (PBS), collected in lysis buffer (25 mM Tris HCl pH 7.5150 mM NaCl, 0.5% Triton X-100 and protease inhibitors), and centrifuged at 20,000*g* for 30 min, and the resultant supernatants collect for the Bradford protein analysis. Protein extracts (100 μg) from HEK or HEK-APP were used for immunoprecipitation experiments. Pull down of endogenous CERT_L_ and APP was performed with 1 μg mAb anti CERT_L_ (3A1-C1) [29] and anti-Aβ mAb 6E10 (Covance), respectively by 1-h incubation at room temperature. mAb anti-syntaxin 6335 (clone 3D10, Abcam) was used as an isotype control. Next, anti-mouse secondary antibodies (Eurogentec) were used to pull down the immune complex. Thereafter, samples were centrifuged at 20,000*g* for 30 min. Pellets were washed three times in 50 μL PBS and boiled in reducing sample buffer containing mercaptoethanol to solubilize immunocomplexes. Then, the proteins were separated on a Tris-HCl 4–15% gradient gel (Bio-Rad) and blotted on nitrocellulose membrane (Millipore). Next, the membranes were probed with anti-Aβ/APP (6E10) or rabbit pAb anti-CERTs (epitope 1–50 of human CERTs, Bethyl Laboratories) antibodies. After 3 washes, membranes were incubated with donkey anti-mouse IRdye 680 and goat anti-rabbit IRdye 800 (Rockland Immunochemicals) and scanned using the Odyssey infrared imaging system (LI-COR Biosciences).

#### Neuronal culture and immunofluorescent staining

Primary neurons were cultured from 5xFAD neonates P0 as described, with modifications [34]. After the cortical area was dissected, the tissue was digested in 0.25% trypsin in Hank’s Balanced Salt Solution (HBSS, Corning) for 15 min. Trypsin activity was stopped with plating medium, DMEM (Gibco, Invitrogen) containing 10% FBS and N2 supplement. Then, the digested tissue was passed through a cell strainer, spun down, and cells resuspended in plating medium. Cells were seeded onto poly-D-lysine-coated coverslips and cultured at 37 °C in a 5% CO_2_ atmosphere. After 4 h, the plating medium was replaced with Neurobasal medium supplemented with B27 supplement, Pen/Strep, and 0.5 mM L-glutamine and kept on for 10–14 days. Every other day, supplemented Neurobasal medium was partially replaced.

Neurons were fixed with 4% PFA in PBS (Thermo Scientific) at 4 °C for 10 min, permeabilized with 0.25% Triton-X in PBS for 5 min, washed three times with PBS, and incubated with 3% BSA for 30 min. Cells were stained with rabbit polyclonal anti-CERTs (epitope 300–350 of human CERTs, Bethyl Laboratories), goat anti-MAP-2 (D-19) (Santa Cruz Biotechnology), and 6E10 anti APP/ Aβ [29]. The following secondary antibodies conjugated to fluorophores were used for detection: anti-mouse IgG Alexa 647, anti-rabbit IgG cy3, and anti-goat IgG Alexa 488. Fluorescence microscopy was performed using Eclipse Ti2-E inverted microscope system (Nikon). Images were processed using Nikon NIS-Elements software equipped with a 3D deconvolution program.

#### Microscale thermophoresis binding analysis

Microscale thermophoresis (MST) analysis was performed in the Monolith NT.155 instrument (Nanotemper). In brief, 20 nM of NT647 labeled CERT was incubated for 20 min at room temperature in the dark with different concentrations of either Aβ_1–42_ (rPeptide Athens) (3–100,000 nM) or control 17 kDa Lama antibody fragment (H6) (1–35,000 nM) in PBS Tween20 (0.01%). Afterward, 3–5 μL of the samples were loaded into glass capillaries (Monolith NT Capillaries, Cat#K002), and the thermophoresis analysis was performed (LED 40.51%, IR laser 80%). Statistical analysis was performed with Origin8.5 Software.

### Aβ aggregation assay and cell-based toxicity assay

#### Transmission electron microscopy (TEM)

Aβ_1–42_, purchased as the lyophilized salt (Bachem), was dissolved in 0.01 M NaOH in ultrapure water to give an Aβ_1–42_ stock solution of *ca* 0.20 mM, which was used immediately to prepare each of the treatments. The remaining peptide stock solution was frozen at − 20 °C until required. Under these highly alkaline conditions, the peptide is fully dissolved and exists only as monomers [35]. Treatments containing Aβ_1–42_ and/or CERT_L_ [29] were prepared in 0.20-μm filtered modified Krebs-Henseleit (KH) medium (118.5 mM NaCl, 4.8 mM KCl, 1.2 mM MgSO_4_, 1.4 mM CaCl_2_, 11.0 mM glucose), buffered in 100 mM PIPES at pH 7.4, including 0.05% w/v sodium azide to inhibit microbial growth. Samples were incubated at 37 °C until their specified time points prior to being prepared onto TEM grids. For replicate samples, Aβ_1–42_ was thawed thoroughly immediately before use and then vortexed briefly. The stock solution was centrifuged at 15,000 rpm for 5 min, and 2.0 μL was then taken ready for concentration determination by absorbance at 280 nm, utilizing a NanoDrop 1000 spectrophotometer (Thermo). The concentration of Aβ_1–42_ was calculated with the Beer-Lambert law and the extinction coefficient 1390 M^−1^ cm^−1^. CERT_L_ concentrations were determined in the same manner with a value of 107,925 M^−1^ cm^−1^ taken as the extinction coefficient using the 72 kDa recombinant CERT_L_ sequence (hCERT_L_, 1875 bp NP_005704.1).

All samples for TEM were prepared via a modified TEM staining protocol [36]. Pre-coated S162 200 mesh formvar/carbon-coated copper grids (Agar Scientific) were inserted into 20.0 μL of the sample beaded onto paraffin film for 60 s, then wicked, passed through ultra-pure water, re-wicked, and placed into 30 μL 2% uranyl acetate (in 70% ethanol), for 30 s. Following staining with uranyl acetate, grids were removed, wicked, passed through ultra-pure water, re-wicked, and placed into 30 μL 30% ethanol for 30 s. Grids were finally re-wicked following this step, covered and allowed to dry for up to 24 h, prior to analysis via TEM.

Samples for TEM were viewed on a JEOL 1230 transmission electron microscope operated at 100.0 kV (spot size 1), equipped with a Megaview III digital camera from Soft Imaging Systems (SIS). Images were obtained on the iTEM universal TEM imaging platform software.

#### Aggregation assay

Aβ_1–42_ was purchased from Anaspec. The peptide was solubilized in sterilized PBS, 0.1% trifluoroacetic acid (TFA) at the concentration 2 mM and frozen in aliquots at − 80 °C. Aliquots were diluted at the final concentration of 20 μM in a total volume of 400 μL containing 1 or 2.5 μM of affinity-purified recombinant CERT_L_ [29]. Samples were kept under rotarod shacking for 1, 2, 4, 8, 12, and 24 h at 37 °C before adding 5 μL to 95 μL of thioflavin T (ThT) concentrated 20 μM, dispensed in 96-well optical plate and measuring fluorescent excitation at 450 nm and emission 486 nm in Victor X3 plate reader (Perkin-Elmer). Two Aβ antibodies against epitope 1–16 (6E10, Biolegend) and 17–24 (4G8, Biolegend) respectively were used to antagonize aggregation at a concentration of 0.1 mg/ml.

#### Toxicity assay

SH-SY5Y cells were seeded on a 96-well plate at a density of 3 × 10^4^ cells per well in 1:1 DMEM:F12 with phenol red, 4 mM glutamine, 200 U/ml penicillin, 200 U/ml streptomycin, MEM non-essential amino acids (100×; Gibco), and 10% FBS and incubated at 37 °C for 24 h, reaching up to 100% confluency, with 5% CO_2_. After 24 h, the medium was removed and replaced with 100 μl/well medium without phenol-red, containing 2% FBS and with 10 μM Aβ_1–42_ oligomers, and/or 1 μM CERT_L_, or alone to control wells and incubated for 24 h. Ten microliters of MTT (4 mg/ml) was added to each well and incubated at 37 °C for 3 h. MTT solution was decanted and the formazan was extracted with 100 μl of 4:1 DMSO:EtOH. Plates were read at 570 nm, with a reference filter at 690 nm.

### Generation of adeno-associated virus

Human collagen type IV alpha 3 binding protein cDNA sequence (hCERT_L_, 1875 bp NP_005704.1) was cloned into the plasmid AAV-6P-SEW a kind gift of Prof. S. Kugler, Department of Neurology, University of Göttingen. The transgene expression was controlled by a human synapsin-1 promoter (hSYN, 480 bp), and an internal ribosome entry site (IRES 566 bp) enabled the co-expression of EGFP [37]. The plasmid expressing exclusively EGFP was used as a control (pAAV-EGFP). The AAV-CERT_L_ plasmid was sequenced by GATC Biotech laboratories and both AAVs plasmids were tested in vitro. AAVs particles were produced as explained previously [38]. In brief, the transfer plasmids pAAV-EGFP or pAAV-CERT_L_ were used to produce AAV2 particles. Eight 15-cm petri dishes each containing 1.25 × 10^7^ HEK 293 T cells in DMEM containing 10% fetal calf serum (FCS) and 1% Pen/Strep (all GIBCO-Invitrogen Corp., New York, NY, USA) were prepared 1 day before transfection. The medium was refreshed 1 h prior to transfection to Iscove’s modified Eagle medium (IMEM) containing 10% FCS, 1% Pen/Strep, and 1% Glutamine. Transfer plasmids were co-transfected using polyethylenimine (PEI, MV25000; Polysciences Inc.) in a ratio of 1:3 with the pAAV-EGFP or pAAV-CERT_L_ resulting in a total amount of 50 μg of plasmid DNA per plate. The day after transfection, the medium was replaced with fresh IMEM with 10% FCS, 1% PS, and 1% glutamine. Two days later (3 days post-transfection), cells were harvested in Dulbecco-PBS (D-PBS, GIBCO) and lysed with 3 freeze-thaw cycles. Genomic DNA was digested by adding 10 μg/ml DNAseI (Roche Diagnostics GmbH) into the lysate and incubated for 1 h at 37 °C. The crude lysate was cleared by centrifugation at 4000 rpm for 30 min. The virus was purified from the crude lysate using the iodixanol gradient method, diluted in D-PBS/5% sucrose and concentrated using an Amicon 100 kDa MWCO Ultra-15 device (Millipore). All AAV vectors were stored at − 80 °C until use. Titers (genomic copies/ml) were determined by quantitative PCR on viral DNA primers directed against the EGFP portion (Forward: GTCTATATCATGGCCGACAA; Reverse: CTTGAAGTTCACCTTGATGC). The AAV particles produced with pAAV-CERT_L_ are referred to in this paper as AAV-CERT_L_ while the particles produced with pAAV-EGFP are named AAV-control.

### Animals

In this study, male mice were used. To investigate transduction efficiency over time, we employed 24 C57BL/6 wild-type (WT) animals. B6/SJL WT and 5xFAD animals were obtained from the Jackson Laboratory and bred in house using 5xFAD x non-carriers. This breeding strategy may breed out the retinal degeneration allele Pde6brd1 from the original strain. The Jackson Lab has observed a less robust amyloid phenotype in this strain. The 5xFAD model carries 5 familial AD mutations, three of them in the human *APP* transgene (Swedish, Florida, and London), and two in the human presenilin-1 (*PS1*) transgene (M146L and L286V mutations). These mutations lead to an increase in Aβ peptide production [32]. Animals were individually housed under a 12 h light/dark cycle in individually ventilated cages. One week before behavioral tests, animals were adjusted to a reversed day-night cycle. Food and water were provided ad libitum throughout the study. All experiments were approved by the Animal Welfare Committee of Maastricht University (project number DEC2013-056 and DEC2015-002) and followed the laws, rules, and guidelines of the Netherlands.

### Stereotactic injection

The animals underwent bilateral stereotactic injections. Mice were placed in a stereotactic head frame, and after midline incision of the skin, two holes were drilled in the skull in the appropriate location using bregma and lambda as references. The layer V of the motor-sensory frontal cortex was targeted; this was verified by light microscopy to observe the dye. Coordinates were determined as follows: anterior-posterior [AP] 0.06, mediolateral [ML] ± 0.15, and dorsoventral [DV] − 0.1 [39]. The AAVs were injected at the dose of 1.12 × 10^8^ transducing unit (t.u.) in the anesthetized mice at a rate of 0.2 μL/min with a final volume of 1 μL for each side.

### Behavioral procedures

The *open field* (OF) task was performed as described elsewhere [40]. Briefly, locomotion activity was assessed in a square divided into 4 equal arenas. At the start of a trial, the animals were placed in the center of each arena. The total distance traveled was measured under low light conditions by a video camera connected to a video tracking system (Ethovision Pro, Noldus).

The *Y-maze spontaneous alternation* (AYM) test was conducted to assess spatial working memory. Mice were placed randomly in one of the three arms of the Y-maze and were left free to explore the arena for 6 min. The number of arm entries and the number of triads were recorded in order to calculate the percentage of alternations to measure working memory.

The *elevated zero-maze* (EZM) was used to measure anxiety. It consists of a circular runway which is divided equally into two opposite open and two opposite enclosed arms. The mice were placed into one of the open arms and allowed to explore the maze over a period of 5 min. The total and relative duration (in %) and distance traveled in the open and enclosed arms were measured in the dark via an infrared video camera connected to a video tracking system (Ethovision Pro, Noldus). Percentage of time spent in the open arms was corrected for latency to first closed arm entry.

The *Y-maze spatial memory* test (SYM) was performed using the same arena as described in AYM above. One arm of the arena was closed by a removable blockade placed in front of it. The mice were placed in one of the open arms, which was randomized over the groups, and allowed to explore the 2 open arms of the maze for 5 min (pre-test). Afterward, the animal was taken from the arena and put back into its home cage. Five hours later, the mouse was placed back into its corresponding start arm of the arena, now with all three arms accessible (post-test). The previously blocked arm was termed the “novel arm”. Memory was evaluated by calculating the amount of time spent in the novel arm corrected for the latency to move from the start arm to another arm and the amount of time the animal spent in the center of the maze [41].

### Immunofluorescence staining

Mice were sacrificed by intracardial perfusion using Tyrode’s solution for the first minute, followed by fixation solution 4% paraformaldehyde (PFA) for 10 min under deep sodium pentobarbital anesthesia (150 mg/kg). The brains were removed and post-fixated overnight in 4% PFA fixation solution and subsequently moved every 24 h in a buffer containing a gradually higher sucrose percentage: 10% and 20% sucrose in 0.1 M PBS. Afterwards, brains were quickly frozen using CO_2_ and dissected into 16-μm-thick sagittal sections using a cryostat (at − 25 °C; Leica). All series of sections were subsequently stored at − 80 °C until further processing. For the CERTs and neuron co-localization stain, we incubated the antibodies separately to reduce the antibody-antigen interaction. Before the antibodies incubation, the slice sections were fixed with acetone 10 min and blocked with 0.3% H_2_O_2_ for 1 h. The sections were incubated with a monoclonal NeuN primary antibody (1:50, chemicon international Inc., Temecula, CA, USA) overnight at 4 °C. Sections were washed 3 times with Tris-buffered saline (TBS), TBS with 0.2% TritonX-100, and TBS. Subsequently, streptavidin Alexa 594 (1:500) applied for 1 h at room temperature. Then, rabbit polyclonal anti-CERTs (epitope 300–350, Bethyl Laboratories) diluted 1:250 was used to detect CERTs. After overnight incubation, and the corresponding secondary antibody Alexa Fluor-647 (1:100) was applied for 1 h at RT. The slices were mounted and stored in 4 °C before taking pictures. Next immunofluorescence co-labeling was performed with either rabbit IgG anti-Iba1 (Wako Pure Chemical Corporation) or mouse IgG anti-glial fibrillary acidic protein (GFAP) combined with human IgG anti-Aβ [29]. Subsequently, the corresponding anti-rabbit or anti-mouse and anti-human secondary antibodies conjugated to Alexa Fluor-594 or 488 (Jackson ImmunoReseach Laboratories) were added for 2 h. Washes were performed 3 times for 10 min in TBS, TBS with 0.2% TritonX-100, and TBS, respectively in between the antibody incubation steps. Densitometric analysis of the stainings was performed on sagittal brain sections at different lateral depth (6–9 sections per animal) with ImageJ. Microglia ramification and sphericity were analyzed as described [42].

### Sphingolipid analysis

#### High pressure liquid chromatography-tandem mass spectrometry (HPLC-MS/MS)

Neuro-2a (N2a) were maintained and prepared for HPLC-MS/MS as described in Supplementary methods. Powder aliquots of cortex, hippocampus, and cerebellum tissue or Neuro-2a (N2a) cell pellet were homogenized in PBS at the concentration of 10 μL/mg. Then, 50 μL of the brain preparation (or 25 μL of plasma) were used to measure Cer, sphinganine (SPA), sphingosine (SPH), and sphingosine-1-phosphate (S1P) as previously described [43, 44]. Briefly, brain preparation (or plasma) was spiked with internal standards mixture prior to undergoing extraction. Data acquisition was done using select ion monitor (SRM) after chromatographic separation and electrospray ionization on the Thermo TSQ Quantum Ultra mass spectrometer (West Palm Beach) coupled with a Waters Acquity UPLC system (Milford) for Cer, sphinganine (SPA), sphingosine (SPH), and sphingosine-1-phosphate (S1P). SM data acquisition was done using multiple reaction monitoring after chromatographic separation and electrospray ionization on the Sciex Qtrap 5500 quadruple mass spectrometer (AB Sciex Inc., Thornhill, Ontario, Canada) coupled with a Shimadzu HPLC system (Shimadzu, Kyoto, Japan). Concentrations of each analyte were calculated against each corresponding calibration curve and corrected for internal standard concentrations.

### Protein extraction

Mice were terminally anesthetized with sodium pentobarbital, perfused, and the brain removed and dissected into the cortex, hippocampus, and cerebellum. Each brain region was then powdered in iron mortar partly emerged in liquid nitrogen, and aliquoted. Frozen tissue from dissected brains was sonicated in about 15 volumes (w/v) of TBS with PhosSTOP and protein inhibitors (Roche). Samples were centrifuged, and the TBS-soluble fraction was aliquoted prior to freezing in liquid nitrogen and stored at − 80 °C in aliquots. The pellet was re-suspended by sonication for 10 s in about 15 volumes of TBS containing 1% Triton-X 100 (TBS-T) and protease inhibitor cocktail. Samples were centrifuged, and the TBS-T-soluble fraction and frozen in aliquots as described for the TBS fraction. The pellet was re-suspended in 70% formic acid to 150 mg/ml based on tissue weight, and mixed by rotation at room temperature for 2 h. Samples were centrifuged, and the formic acid-soluble fraction was neutralized (with 20 volumes of 1 M Tris base) and frozen in aliquots at − 80 °C. Total protein content in the TBS and TBS-T extractions was determined with Bio-Rad DC (Life Science Group) protein assay following the manufacturer’s instructions.

### Aβ immunoassay and Western blot

#### Immunoassay

Microplates Microlon/F-shape REF 655092 (Greiner) were coated with 1 μg/mL of human 3D6 [29] overnight at room temperature in coating buffer (sodium carbonate pH = 9.6 0.05 M NaCO_3_ in MQ water). After washing plates (washing buffer 0.05% Tween- 20 in PBS were blocked with 4% not fat dry milk and incubated with brain homogenates or with Aβ to generate the standard curve. Next plates were washed, incubated with 50 ng/mL of biotinylated human 20C2 [29], and washed again. Finally, plates were incubated with streptavidin-HRP (Jackson ImmunoResearch Laboratories) diluted 1:8000 and developed using 3,3′,5,5′-Tetramethylbenzidine (TMB). The absorption was measured at 450 nm within 30 min of stopping the reaction with 2 M H_2_SO_4_ using the Perkin Elmer 2030 manager system.

#### Western blot

TBS and TBS-T samples corresponding to 40 μg of total protein we loaded onto a precast TGX 4–16% gel (Bio-Rad). Then, samples were transferred to PDVF membranes, blocked with Odyssey blocking buffer (LI-COR Bioscience) and probed with 1 μg/mL mAb anti-Aβ (6E10, Covance). After incubation with donkey anti-mouse IRdye680 (Rockland Immunochemicals) diluted 1:1000 in Odyssey blocking buffer, the membrane was scanned and analyzed with the Odyssey imager. The intensities of the APP bands detected at 100 kDa were measured with the Odyssey imager.

### Reverse transcription polymerase chain reaction (RT-PCR)

Total RNA was isolated from 20 to 50 mg of cortex using Trizol reagent (Invitrogen). One microgram of total RNA was treated with DNAse I and transcribed into cDNA (Superscript III, Invitrogen). PCR was performed in duplicate with SensiMix™ SYBR® Low-ROX Kit (Bioline) using the set of primers reported in supplementary Table 1. Fold changes of expression relative to control were determined after normalization to GAPDH and Actin. Fold change was calculated by the comparative Ct method [45].

### Statistical analysis

Statistical analysis was performed using the Statistical Package for the Social Sciences (SPSS 23.0 SPSS Inc., Chicago, IL, USA). All graphs were designed in GraphPad Prism (version 5, GraphPad Software, San Diego, CA, USA). All data shown are expressed as mean ± standard error of the mean (SEM). Behavioral tasks, SLs, RT-PCR, and cytokines data were analyzed with two-way analysis of variances (ANOVA) with AAV treatment and genotype as independent factors. Least significant difference (LSD) was used for post hoc testing. Fluorescence amyloid-β aggregation assays were analyzed with repeated measure ANOVA or ANOVA followed by Dunnett’s multiple comparisons test. Comparison of mean values from two groups was performed by an unpaired two-tailed Student’s *t* test or by Mann–Whitney *U* test for non-parametric testing. *P* values were considered as significant if *p* ≤ 0.05 and marked with (*). Results were marked with (**) if *p* ≤ 0.01 or (***) if *p* ≤ 0.001.

## Results

### CERT_L_ directly affects Aβ aggregation and toxicity in vitro

**Fig. 1:**
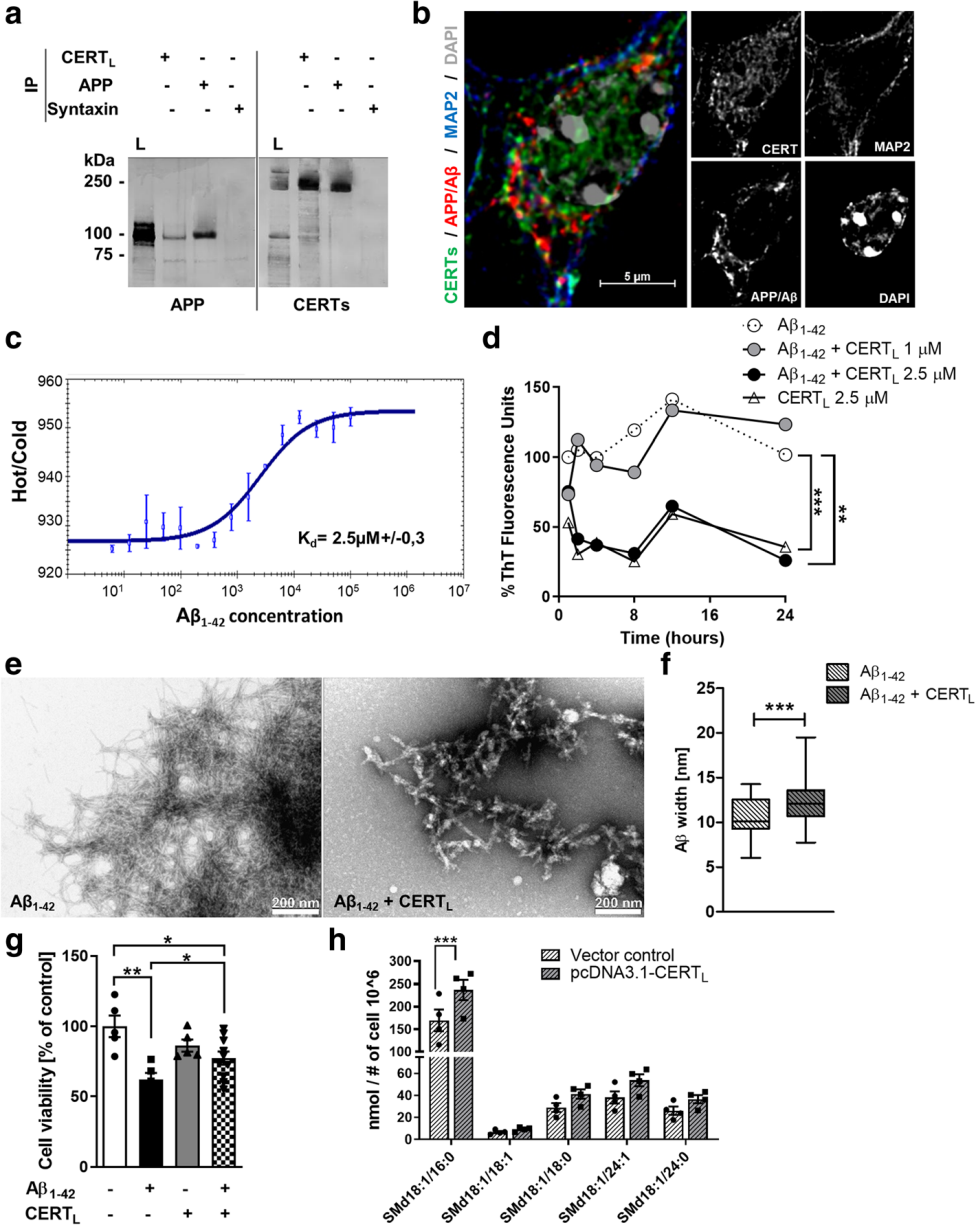
CERT_L_ binds directly to APP and Aβ and reduces Aβ aggregation and toxicity in vitro. **a** Protein interaction detected using co-IP of APP and CERT_L_ in HEK-APP. The total cell lysate of HEK-APP cells (L) lane 1 and the total cell lysate of HEK-APP cells IP using APP/Aβ (lane 2), CERT_L_ (lane 3), and syntaxin (isotype control) (lane 4) antibodies were analyzed by Western blot. APP (1) and CERTs proteins were detected. The isotype control syntaxin protein was negative. Molecular weight markers are indicated (kDa). **b** Immunofluorescent staining showing co-localization of CERTs and APP/Aβ in primary neurons isolated from 5xFAD brains. Neuronal marker MAP 2 was used to immuno-label neuronal cells. DAPI was used for nuclei staining. Scale bar 5 μm. **c** CERT_L_ and Aβ_1–42_ interaction was measured by microscale thermophoresis. The dissociation constant (*K*_d_) calculated was 2.5 ± 0.3 μM. **d** Percentage of Thioflavin T (ThT) fluorescence intensity to detect Aβ_1–42_ 20 μM aggregation in the absence or presence of recombinant CERT_L_ 1 or 2.5 μM at different time points. Each data point represents the percentage of mean fluorescent intensity of three wells (repeated measures ANOVA; Dunnett’s multiple comparisons test **p < 0.01, ***p < 0.001). **e**, **f** TEM analysis of 2 μM Aβ_1–42_ aggregation in the absence and presence of 0.1 μM CERT_L_ showed a different aggregation pattern quantified by Aβ width (Student’s *t* test ***p < 0.001). **g** Measurement of cell metabolic activity of SH-SY5Y by MTT assay in cells incubated with medium alone (control) or medium containing 10 μM Aβ_1–42_, Aβ_1–42_, and 1 μM CERT_L_, or CERT_L_ alone for 24 h. Graph bar expressed as means ± S.E.M % of control *N* = 5–10 (one-way ANOVA, Bonferroni correction *p < 0.05; **p < 0.01). **h** SM d18:1/16:0, SM d18:1/18:1, SM d18:1/18:0, SM d18:1/24:1, and SM d18:1/24:0 measured by HPLC-MS/MS in N2a cells after 48-h transfection with vector control or pcDNA-CERT_L_. Graph bar expressed as means ± S.E.M % of control *N* = 4/group (one-way ANOVA, Holm-Sidak’s multiple comparisons test ***p < 0.001)

It has been demonstrated that specific forms of CERTs can be released extracellularly or found membrane bound [30]. We have shown that CERTs can be found in proximity to Aβ plaques in AD brain, where they partially co-localize with SAP and with amyloid fibrils [29]. Here, we tested whether the long isoform of CERT, CERT_L,_ interacts with Aβ and its precursor protein APP. Cellular extracts of HEK-APP, expressing endogenous CERT_L_, and stably expressing APP, were incubated with (mouse) monoclonal antibodies (mAbs) against CERT_L_, APP, or syntaxin 6 (used as a negative control) for immunoprecipitation (IP). Pull-down was performed with anti-mouse antibodies for all 3 conditions. Western blot analysis of the samples showed efficient direct IP of CERT_L_ and APP by their respective antibodies. APP was detected as a band of ~ 100 kDa whereas CERT_L_ was detected as a band of ~ 200 kDa (Fig. 1a, left panel). As a result of co-immunoprecipitation, these two respective bands were also found when either CERT_L_ or APP were pulled down, but not when syntaxin 6 was used instead (Fig. 1a, right panel). The results, representative of three independent experiments, showed an interaction between CERT_L_ and APP/Aβ confirming our previous pull down of APP with CERT in the brain lysate of AD animals manifesting severe Aβ pathology [29]. The two proteins showed partial colocalization in primary neurons of 5xFAD mice at the plasma membrane and in the perinuclear region (Fig. 1b). This was also observed in brain sections of 5xFAD immunolabeled for CERTs and APP/Aβ (Supplementary Figure 1A). As shown by microscale thermophoresis (MST) analysis, CERT_L_ also bound to Aβ_1–42_ (K_d_ = 2.5 μM) (Fig. 1c) and by Western blot (Supplementary Figure 1B and C). Having shown that CERT_L_ directly binds to APP and Aβ, we next tested whether binding of CERT_L_ to Aβ_1–42_ could directly influence the spontaneous fibrillization of Aβ_1–42_ by thioflavin T (ThT) fluorescence spectrometry and TEM. In the absence of CERT_L_, ThT fluorescence of Aβ_1–42_ peaked after 10–12 h indicating amyloid formation. With the addition of CERT_L_ (2.5 μM) Aβ_1–42_ maximum ThT fluorescence reduced of about 75% around 24 h. In contrast, at 1 μM concentration, CERT_L_ was ineffective at preventing Aβ_1–42_ fibrillization (Fig. 1d). Fibrillization was also blocked by Aβ antibodies (Supplementary Figure 1D).

In addition to the ThT fluorescence assay, we studied Aβ aggregation in the presence of CERT_L_ by TEM. The TEM images showed that amyloid-like fibrils were observed in both conditions with and without CERT_L_ in combination with Aβ. However, the amyloid structure identified in the presence of CERT_L_ was not linear in shape and the fibril width varied significantly (Fig. 1e, f) when compared to Aβ_1–42_ alone. The aggregates observed with CERT_L_ plus Aβ_1–42_ could be precursors to amyloid fibril formation, but not classic straight amyloid fibril formation as previously reported [46].

Furthermore, using SHSY-5Y cells, we examined the effect of CERT_L_ on cell viability when Aβ_1–42_ was present. The addition of oligomeric Aβ_1–42_ to the culture medium of SHSY-5Y cells resulted in a 38% decrease in viability after 24 h, as measured by MTT reduction relative to control conditions (*p* < 0.01). Interestingly, the simultaneous addition of CERT_L_ significantly ameliorated the toxic effect of Aβ_1–42_ (Fig. 1g). Our results indicate that CERT_L_ forms complexes with APP and Aβ. The interaction of CERT_L_ with Aβ affected spontaneous aggregation and toxicity of the peptide.

As aforementioned, CERTs are important regulators of cellular Cer and SM balance. For this reason, we investigated the effect of modulating CERT_L_ levels on SL composition in vitro. After 48-h cell transfection with pcDNA3.1 driving expression of CERT_L_, SM d18:1/16:0 was significantly increased while Cer were unchanged (Fig. 1h). Furthermore, CERT_L_ overexpression did not affect cell viability (data not shown).

### Ceramide species are increased in 5xFAD compared to WT animals depending on brain region and acyl chain length

**Fig. 2:**
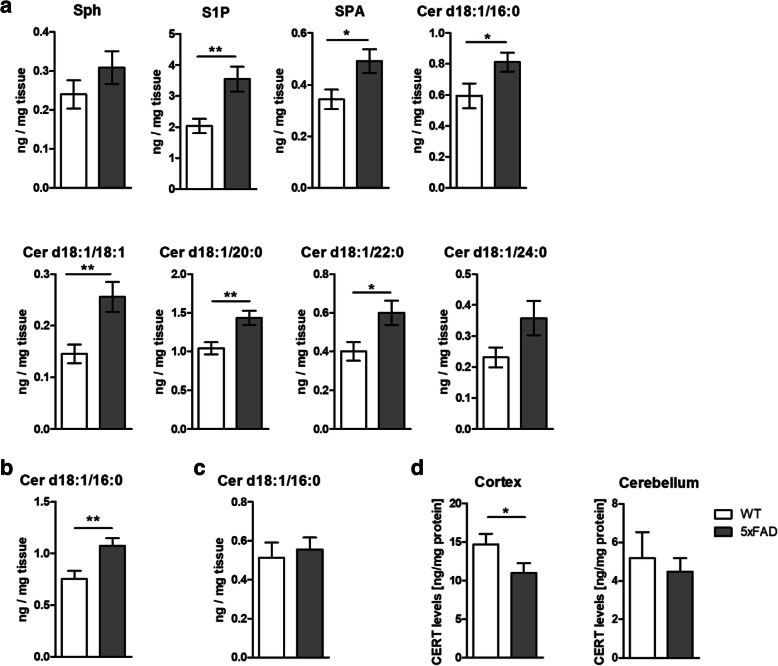
Ceramide levels are increased in 5xFAD compared to WT animals depending on brain region and acyl chain length while CERT levels are reduced. Sphingolipids levels were measured in the hippocampus (**a**), cortex (**b**), and cerebellum (**c**) by HPLC-MS/MS of WT and 5xFAD mice. Sphingolipids were classified based on acyl chain number of carbons (Sph, S1P, SPA, Cer d18:1/16:0, Cer d18:1/18:1, Cer d18:1/20:0, Cer d18:1/22:0, and Cer d18:1/24:0). CERT was quantified by ELISA in protein extract of cortex and cerebellum of WT and 5xFAD animals. Bars represent the mean ± S.E.M per group *N* = 11–12 (Student’s *t* test *p < 0.05; **p < 0.01)

The 5xFAD model carries 5 familial AD mutations. These mutations lead to a rapid increase of Aβ peptide production. By 6 weeks of age, mice display elevated levels of Aβ, amyloid deposits, and age-dependent amyloid pathology accompanied by increase of inflammatory marker levels in the CNS [47–50]. However, it is unknown if this model shows also an increase of Cer level in the brain as it has been reported in the brains of AD patients [13–16]. Sphingolipid species were determined with HPLC-MS/MS in the cortex, hippocampus, and cerebellum of 5xFAD and wild-type (WT) male mice at 25–26 weeks of age (Supplementary Table 1 reports complete analysis). The analysis showed a significant elevation of Cer d18:1/16:0 levels in the cortex and of sphinganine (SPA), S1P, Cer d18:1/16:0, Cer d18:1/18:1, Cer 18:d1/20:0, and Cer d18:1/22:0 in the hippocampus of the 5xFAD animals compared to WT mice (Fig. 2a, b). In the cerebellum, only S1P levels were found to be significantly higher in the 5xFAD animals compared to controls. These results indicate that at that specific age and disease stage of the animals, the cortex and the hippocampus are more susceptible to increase of Cer levels while the cerebellum is less affected. This suggests that the increase of Cer levels correlate with amyloid burden. In line, the cortex and hippocampus are the areas where the plaques are first reported to appear in this model [32]. Quantification of CERT levels in the cortex by immunoassay showed a significant reduction of the protein concentration in AD brains compared to controls at 25–26 weeks of age [51]. CERTs reduction was not observed in cerebellum (Fig. 2d). Our data suggest that SL metabolism is shifted towards increased production of Cer at different degrees depending on the brain region. Furthermore, CERT concentration was reduced in the cortex of 5xFAD mice compared to WT.

### AAV-mediated neuronal expression of CERT_L_ in mouse brain

**Fig. 3:**
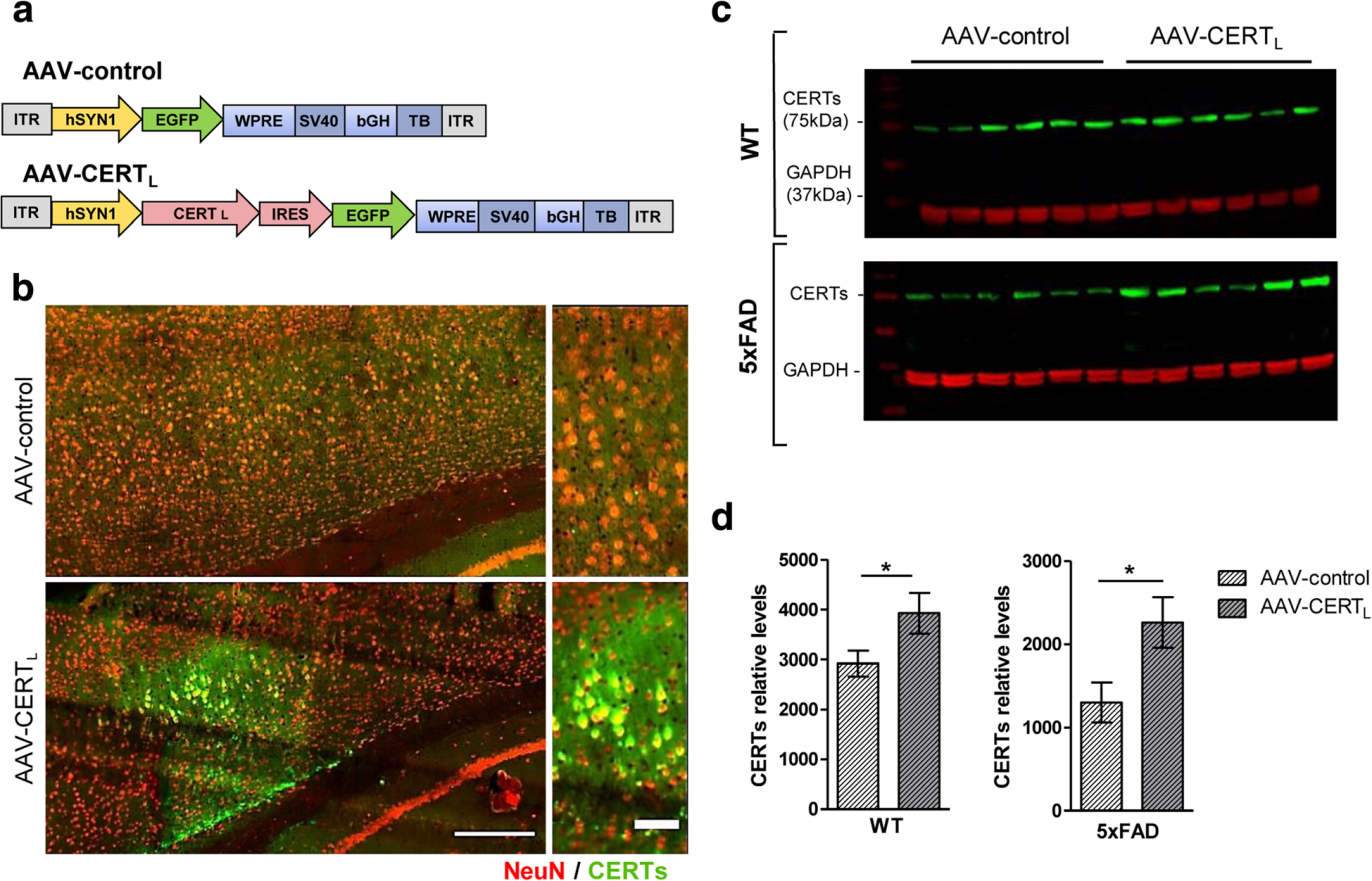
AAV-mediated neuronal expression of CERT_L_ in mouse brain. **a** The recombinant genomes of the two AAV-2 vectors. ➔ Abbreviations: From left to right, ITR, inverted terminal repeats; hSYN1, human synapsin 1 gene promoter; CERT_L_, cDNA sequence coding for ceramide transfer protein long isoform (hCERT_L_, 1875 bp NP_005704.1); IRES, internal ribosome sequence for translation initiation; EGFP, cDNA coding for enhanced green fluorescent protein (GFP); WPRE, woodchuck hepatitis virus posttranscriptional control element; bGH, bovine growth hormone gene-derived polyadenylation site; TB, synthetic transcription blocker. **b** Representative images of immunofluorescent staining of cortical brain area from 5xFAD animals treated with AAV-control or AAV-CERT_L_. Section was co-stain for CERTs protein (green), neuronal marker NeuN (red). Scale bar 200 μM and 50 μM. **c** Western blot showing band intensities of CERTs and GAPDH. **d** Relative quantification of CERTs levels normalized to GAPDH in cortical protein extract from WT and 5xFAD animals treated with AAV-control or AAV-CERT_L._ Bars represent mean one representative experiment with 5–7 samples *per* group (Mann–Whitney test, *p < 0.05)

To test the hypothesis that increasing CERT_L_ levels in the cortex counteract the SL dysbalance in 5xFAD animals and Aβ formation, we generated AAV2 particles carrying the CERT_L_ cDNA sequence controlled by the neuron-specific synapsin promoter (Fig. 3a). The AAV-CERT_L_ was tested in vitro on cortical rat primary cell culture, proving to effectively transduce neurons (Supplementary Figure 2A). Next, 8-week-old WT animals underwent stereotactic surgery and AAV-CERT_L_ or AAV-control were injected in the layer V of the motor cortex (Supplementary Figure 2B). The transduction efficiency was evaluated 1, 2, 6, and 12 weeks post-injection by immunohistochemistry (Supplementary Figure 1C-D) and by RT-PCR (Supplementary Figure 1E). The AAV-CERT_L_ was shown to effectively transduce neurons and expressed the CERT_L_ protein for at least 12 weeks. The AAVs were then injected in 5xFAD mice in a similar fashion. Mice underwent stereotactic surgery for CNS administration of AAV particles at 12–13 weeks of age and were monitored for 12 weeks. Layer V of the frontal cortex was targeted since the Aβ accumulation is the most severe there [32] and CERT levels are reduced in this mouse model. After 12 weeks CERT overexpression was confirmed by immunofluorescence (Fig. 3b) and by Western blot analysis (Fig. 3c, d). The immunolabeling of CERTs, with neuronal marker NeuN, showed colocalization in the layer V of the motor cortex. Relative quantification of CERTs levels in cortex homogenates by Western blot illustrated a significant increase of CERTs levels in AAV-CERT_L_-treated groups (Fig. 3d).

### 5XFAD and WT mice injected with AAV-CERT_L_ and AAV-WT did not show behavioral abnormalities

**Fig. 4:**
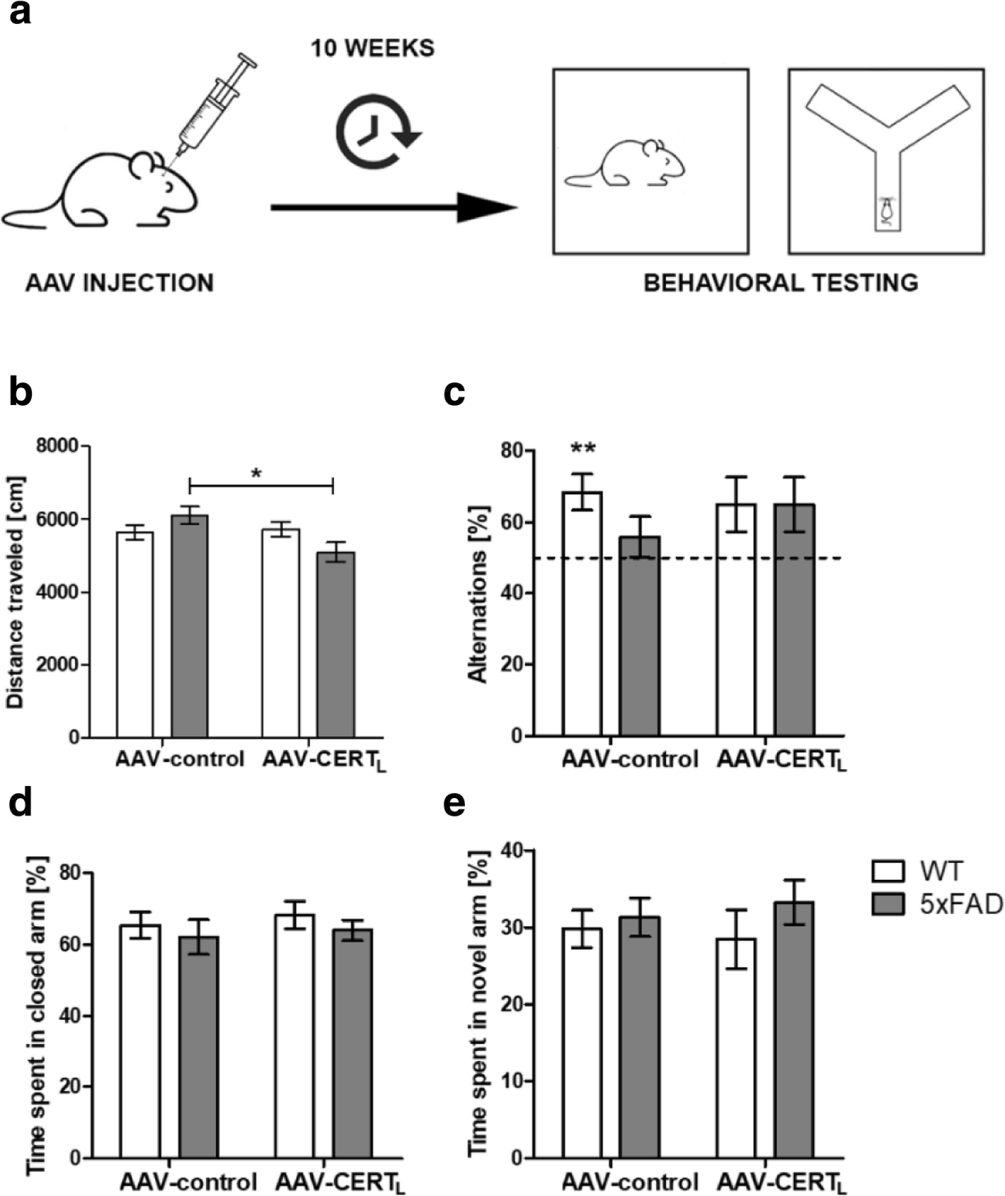
No behavioral abnormalities 10 weeks after injection of AAV-CERT_L_. **a** The effects of CERT_L_ over-expression were investigated in 30 5xFAD and 30 WT males. Mice were bilaterally injected at 12–13 weeks of age with AAV-CERT_L_ or AAV-control particles at the dose 1.12 × 10E8 transducing unit (t.u.). Starting at week 22 of age, animals were challenged with the following behavioral tests: open field (OF) for locomotion activity, alternate Y-maze (AYM) and spatial Y-maze (SYM) for spatial memory, and elevated zero-maze (EZM) for anxiety. **b** Locomotion expressed as distance traveled in OF task. **c** The graph shows the results of the working memory in the AYM task as a percentage of correct alternation in the first four triads. Percentage were compared to 50% chance levels (one sample *t* test **p < 0.01). **d** Anxiety was assessed, measuring the percentage of time spent in the closed arm in EZM. **e** Memory was measured in SYM expressed as a percentage of time spent in the novel arm. Bars represent the means ± S.E.M per group *N* = 10–20 (two-way ANOVA, interaction effect *F* = 4.170 *p* = 0.0463, LSD, *p < 0.05)

At week 10 post-injection, mice were challenged with a behavioral test battery in the following sequence: open field (OF) for assessing locomotion activity, Y-maze spontaneous alternation (AYM), and SYM for assessing spatial memory and elevated zero-maze (EZM) for examining anxiety (Fig. 4a–e). It has been reported that 5xFAD mice exhibit changes in hippocampus-dependent spatial working memory by 16 to 24 weeks of age [32]. However, in our hands, no difference was found in the performance of 5xFAD compared to WT in locomotion (Fig. 4b), memory (Fig. 4c, e), and anxiety (Fig. 4d). Our data showed that the WT animals treated with the control virus performed above the 50% chance level in the AYM (Fig. 4c). Furthermore, in the OF, the AAV-CERT_L_ attenuated 5xFAD hyperactivity, even though 5xFAD treated with AAV-control did not perform significantly different from WT animals (Fig. 4b, two-way ANOVA, interaction *F* = 4.170, *p* = 0.0463). Overall, these data suggest that no detrimental behavioral effects in the animals were observed after CERT_L_ over-expression.

### AAV-CERT_L_ decreased Cer d18:1/16:0 and increased SM levels in the cortex

**Fig. 5:**
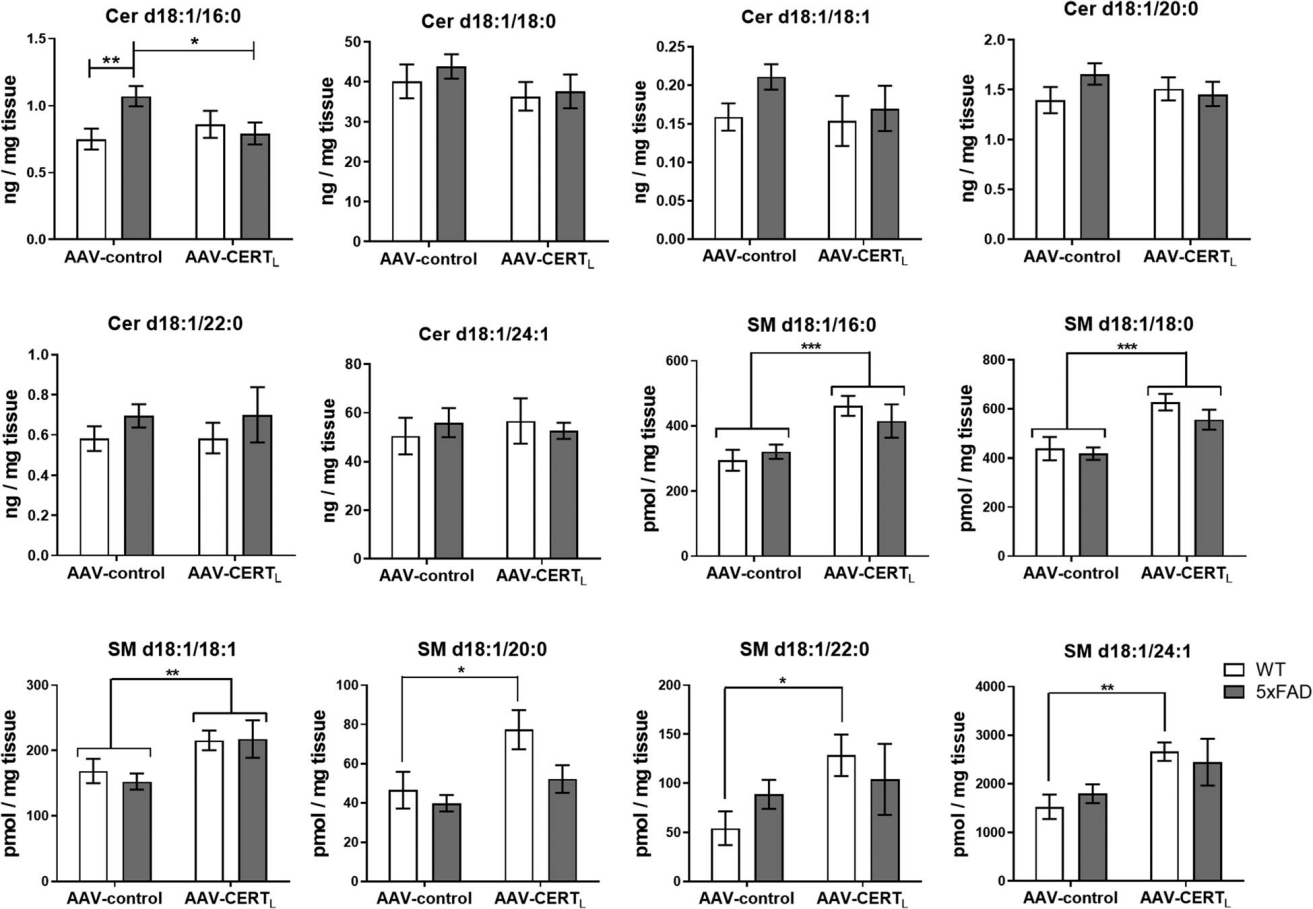
AAV-CERT_L_ reduces Cer d18:1/16:0 and increases sphingomyelin species in the cortex. Sphingolipids levels were measured in the cortex by HPLC-MS/MS. Ceramides were classified based on acyl chain number of carbons (Cer d18:1/16:0, Cer d18:1/18:0, Cer d18:1/18:1, Cer d18:1/20:0, Cer d18:1/22:0, and Cer d18:1/24:1) as well as sphingomyelin (SM d18:1/16:0, SM d18:1/18:0, SM d18:1/18:1, SM d18:1/20:0, SM d18:1/22:0, and SM d18:1/24:1). Ceramide levels were expressed as ng/mg tissue, while sphingomyelins were expressed as pmol/mg tissue. Bars represent the mean ± S.E.M per group *N* = 5–12 (two-way ANOVA, LSD, significant effects, *p < 0.05; **p < 0.01; ***p < 0.001)

One of the main functions of CERTs is to shuttle Cer from the ER to the Golgi [23]. It has been reported that toxic increase of Cer species in the muscle can be attenuated by overexpressing hCERT cDNA [52]. HPLC-MS/MS analysis of brain cortex tissue revealed a significant reduction of Cer d18:1/16:0 level due to CERT_L_ overexpression (*p* < 0.05). This effect was not observed in WT animals but only in 5xFAD animals where Cer d18:1/16:0 level was significantly elevated (*p* < 0.01). This transport of Cer to the Golgi is crucial for the de novo synthesis of more complex SLs such as SM. Previous data from in vitro experiments reported that blocking CERTs function SM levels would significantly decrease [27]. Hence, if CERTs activity is enhanced, we expected an increase in SM levels. As reported above, we found that in vitro overexpression of CERT_L_ increased the levels of certain species of SM. SL analysis of the cortex showed that the levels of most of SM species (SM d18:1/16:0 *p* < 0.001, SM d18:1/18:0 *p* < 0.001, SM d18:1/18:1 *p* < 0.01, SM d18:1/20:0 *p* < 0.05, SM d18:1/22:0 *p* < 0.05, and SM d18:1/24:1 *p* < 0.01) were increased in AAV-CERT_L_ treated animals (Fig. 5). The only SM species not found significantly elevated was the SM d18:1/24:0, whose precursor Cer d18:1/24:0 has been reported to be poorly transferred by CERTs [53].

These data suggest that CERT_L_ overexpression is effective in reducing Cer increase by intensifying the transfer to the Golgi, which leads to an increase of SM. While the Cer attenuation was restricted to a pathological increase of Cer d18:1/16:0 level, SM elevation was consistent in all AAV-CERT_L_-treated animals. This shift in SL composition did not affect apoptosis markers in the cortex quantified by RT-PCR (Supplementary Figure 6A).

### AAV-CERT_L_ reduces Aβ levels by decreasing APP cleavage in the cortex of 5xFAD mice

**Fig. 6:**
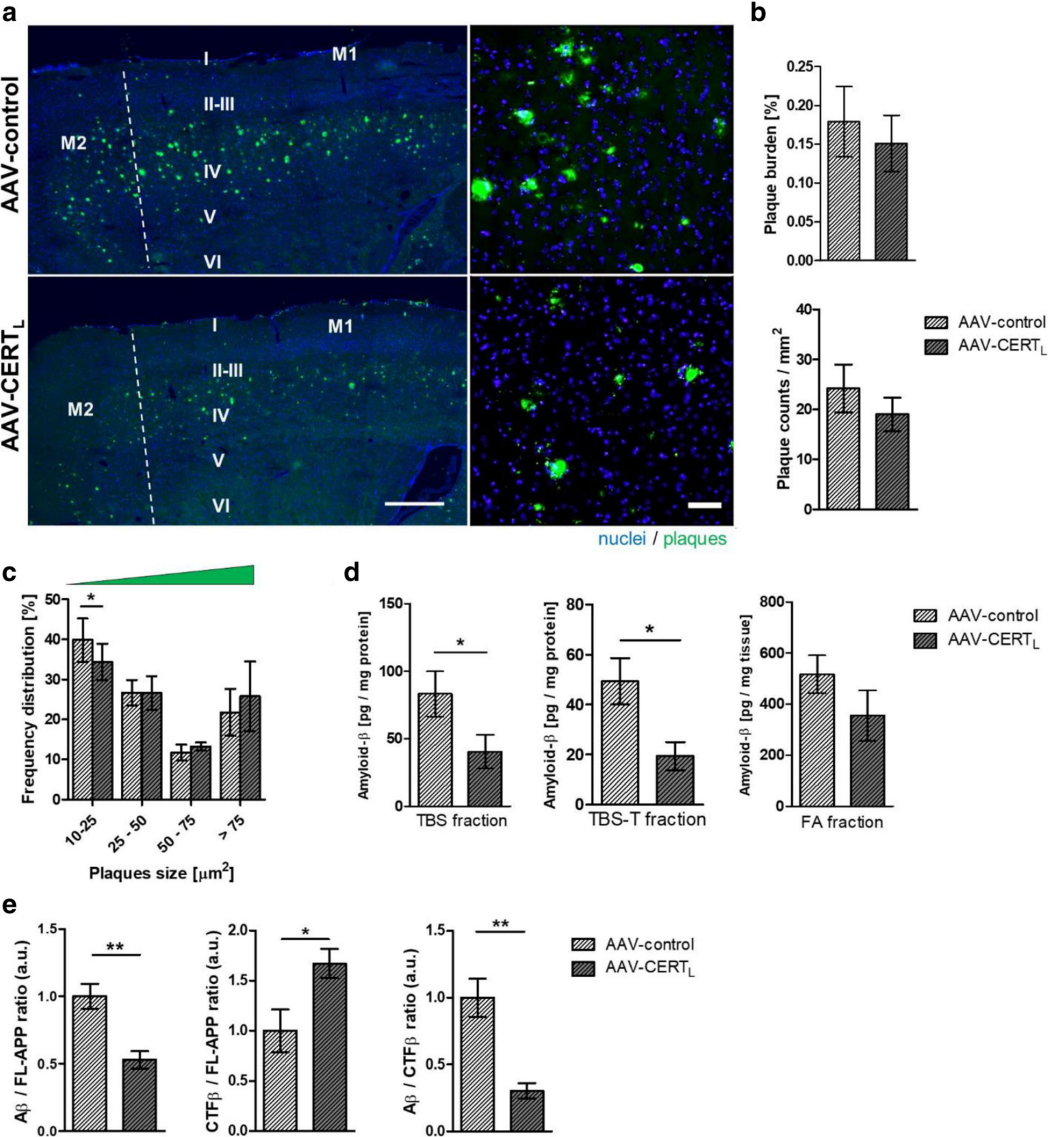
Neuronal increase of CERT_L_ reduces Aβ by decreasing APP cleavage. **a** Representative photomicrographs of sagittal brain sections imaging the motor sensory cortex (M1 and M2) stained for nuclei in blue and Aβ plaques in green. All photomicrographs were exposed and processed identically. Scale bar represents 200 and 50 μm (from right to left). **b** Immunofluorescent quantification of plaques measured by the percentage of area, plaques counts/mm^2^. **c** Frequency distribution of plaques based on size (10–25 μm) (error bars represent ± SEM of 4–6 animals per experimental condition, ANOVA, Bonferroni correction, significant effects, *p < 0.05; **p < 0.01). **d** Aβ quantification in three extraction buffers, BS, TBS-T, and formic acid (FA) by ELISA showed that Aβ was significantly reduced in the soluble fractions in the cortex but not in the insoluble fraction (Student’s *t* test *p < 0.05). **e** Western blot analysis of TBS cortex homogenate stained with 6E10 antibody showed that ratios of amyloid Aβ/FL-APP and Aβ/CTFβ are reduced while CTFβ/FL-APP is increased in AAV-CERT_L_-treated animals while CTFβ/FL-APP is increased. Error bars represent ± SEM of 5 animals per experimental condition (Student’s *t* test *p < 0.05; **p < 0.01) (full length amyloid precursor protein = FL-APP; amyloid-β peptide = Aβ; C-terminal fragment β = CFTβ). Western blot membranes are shown in Supplementary Figure 5

Since our previous data indicated that CERT_L_ could be released to the extracellular milieu and directly affects Aβ aggregation and toxicity in vitro, we investigated the effect of CERT_L_ overexpression on Aβ deposition. (Fig. 6a). Statistical analysis showed no significant difference in plaque load between the 5xFAD groups at 24–26 weeks of age (Fig. 6b). However, the percentage of small plaques size (10–25 μm) was reduced (*p* < 0.05) in AAV-CERT_L_-treated 5xFAD brains (Fig. 6c). Furthermore, Aβ quantification of brain homogenate in TBS soluble and TBS-T soluble fraction showed that Aβ levels were reduced in samples treated with AAV-CERT_L_ (*p* = 0.04 and *p* = 0.03, respectively), while in the formic acid soluble fraction no change was observed (*p* = 0.29) (Fig. 6d). Since it has been reported that APP cleavage can be affected by Cer composition [21, 22], we investigated if the reduction of Cer and increase of SM levels mediated by AAV-CERT_L_ is associated with altered processing of APP. The ratio Aβ/FL-APP was decreased in CERT_L_ overexpressing mice implying a reduction of Aβ biogenesis or increased clearance of Aβ (*p* < 0.01). Since the CTFβ is the product of β-secretase cleavage of APP and the immediate precursor of Aβ formation [54], the ratio of CTFβ/FL-APP bands’ intensities was used to assess APP processing by β- and γ-secretase. The CTFβ/FL-APP was found an increase in AAV-CERT_L_ treated 5xFAD animals (*p* < 0.05) (Fig. 6e). Meanwhile, the ratio Aβ/CTFβ was reduced in brains of CERT_L_ overexpressing mice (*p* < 0.01) (Fig. 6e and Supplementary Figure 5). These results indicate that AAV-CERT_L_ affects the proteolytic processing of APP by β- and/or γ-secretase.

These results show that a specific balance of Cer to SM and/or interaction of CERT_L_ with APP is critical for APP cleavage and Aβ biogenesis.

### Four weeks of administration of CERTs inhibitor (HPA-12) exacerbates Cer and Aβ pathology in AD transgenic mice

**Fig. 7:**
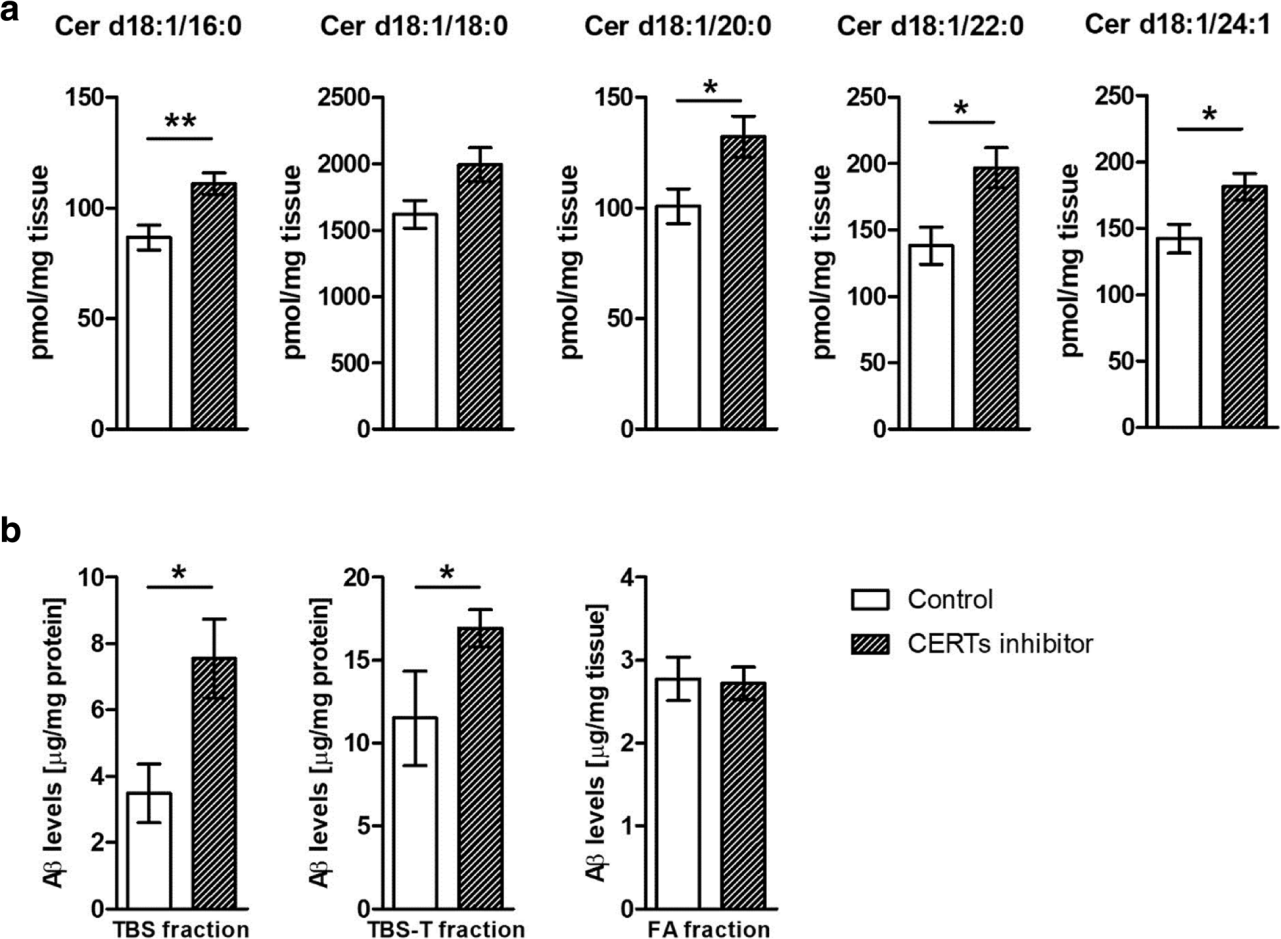
CERTs inhibitor increases Cer and Aβ levels in the brain of transgenic AD mice. **a** Sphingolipid levels were measured in the cortex by HPLC-MS/MS. Ceramides were classified based on acyl chain number of carbons (Cer d18:1/16:0, Cer d18:1/18:0, Cer d18:1/22:0, and Cer d18:1/24:1) and levels were expressed as pmol/mg tissue. Bars represent the mean ± S.E.M per group *N* = 10–12 (Student’s *t* test *p < 0.05) Aβ quantification in hippocampus homogenate extracted in three buffers, TBS, TBS-T, and formic acid (FA) by enzyme-linked immunoassays. Means of each fraction were compared with unpaired *t* test (control *N* = 10; HPA-12 *N* = 13; *p < 0.05)

To test if efficient Cer trafficking from the ER to the Golgi is vital in the regulation of the Cer levels and Aβ formation, we administered the CERTs inhibitor N-(3-hydroxy-1-hydroxymethyl-3-phenylpropyl) dodecanamide (HPA-12) for 4 weeks to AD transgenic mice. As expected Cer d18:1/16:0, Cer d18:1/20:0, Cer d18:1/22:0, and Cer d18:1/24:1 levels were found an increase in the brain (Fig. 7a). Moreover, Aβ levels were increased by 117% in the TBS soluble fraction (*p* < 0.05) and by 47% in the TBS-T soluble fraction of brain homogenates (*p < 0.05) of CERTs inhibitor-treated AD animals. No significant changes were found in the FA insoluble fraction (Fig. 7b).

These data suggest that efficient Cer transfer from ER to *trans*-Golgi is critical to control Cer levels and thereafter APP processing. Pharmacogical interference with CERT activity augments Cer levels and increases substantially Aβ biogenesis and/or fibrillization.

### AAV-CERT_L_ reduces microglia immunoreactivity as shown by Iba1 labeling and CD86 expression

**Fig. 8:**
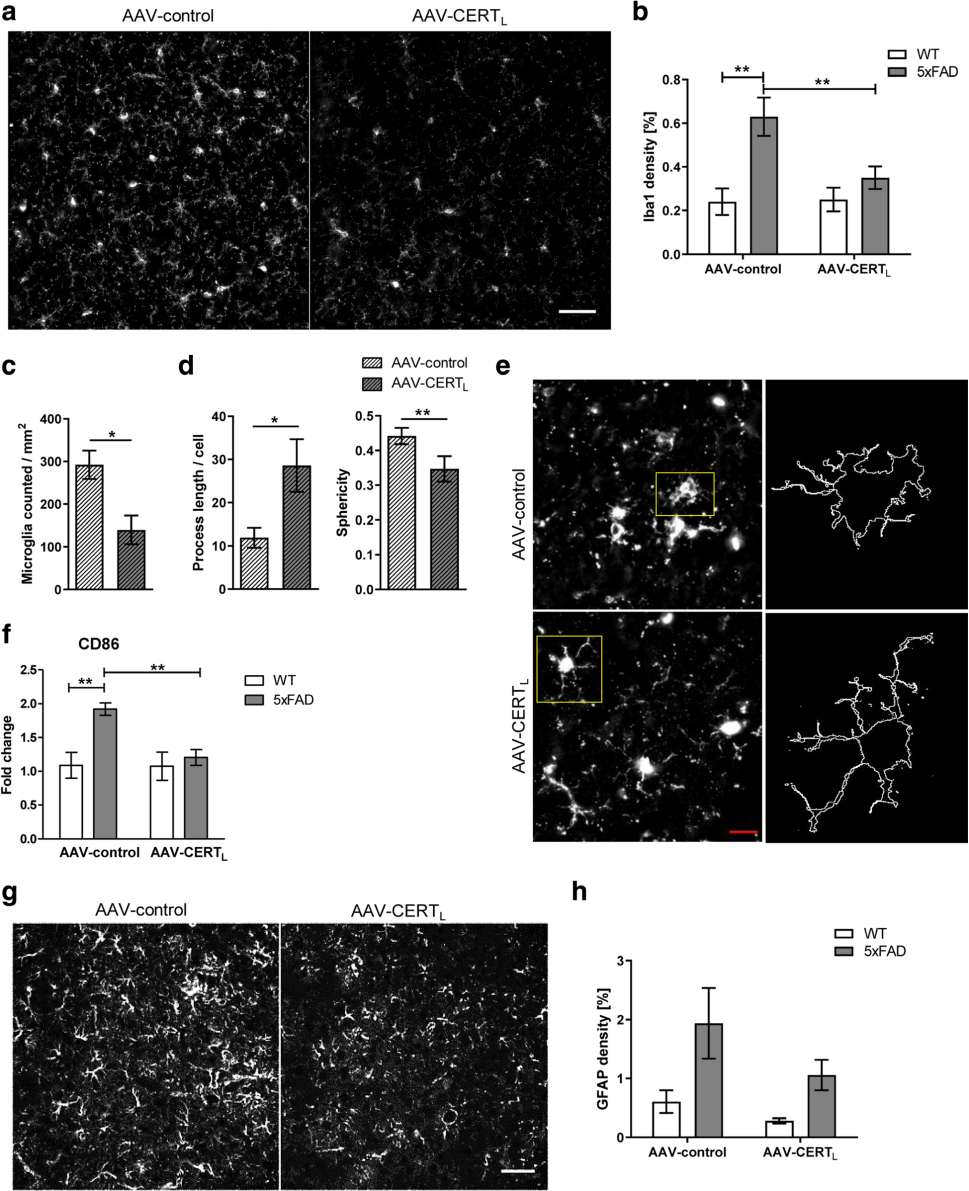
AAV-CERT_L_ reduces microglia reactivity to Iba1 and CD86 expression levels but has no significant effect on GFAP immunoreactivity in the cortex of 5xFAD mice. **a** Representative photomicrograph of Iba1 staining in the cortical motor sensory region of 5xFAD animals treated with AAV-control or AAV-CERT_L_ (scale bar 50 μm). **b** Densitometric analysis of Iba1 staining represented as a percentage of the area (AAV-control *n* = 6 and AAV-CERT_L_ *n* = 4 for WT and 5xFAD groups). **c** Densitometric analysis of Iba1 staining represented as number of positive Iba1 cells/mm^2^ (AAV-control *n* = 6 and AAV-CERT_L_ *n* = 4 for 5xFAD groups). **d** Length of microglia ramification and sphericity per cell in AAV-control or AAV-CERT_L._ Morphological analysis was performed on 3–5 pictures/group. **e** Illustrations of the microglia morphological analysis applied to a fluorescent photomicrograph captured with × 60 objective with a single cell cropped to show details. Scale bar = 20. **f** Analysis of gene expression of membrane markers CD86 (4–5 number of animals per group). **g** Representative photomicrographs of GFAP staining in the cortical motor sensory region of 5xFAD animals treated with AAV-control or AAV-CERT_L_ (scale bar 50 μm). **h** Densitometric analysis of GFAP staining represented as a percentage of the area (AAV-control *n* = 6 and AAV-CERT_L_ *n* = 4 for WT and 5xFAD groups). Bars represent the mean ± S.E.M per group (two-way ANOVA, LSD, significant effects, *p < 0.05; **p < 0.01)

We have observed that CERTs are associated with Aβ plaques in AD brain where microglia cells are engaged [29]. Thus, we investigated if microglia cells were affected by CERT_L_ overexpression in the cortex. To achieve this aim, brain sections were analyzed for Iba1 reactivity. AAV-CERT_L_-treated brains had a decreased immunoreactivity to Iba1 (percentage of area *p* < 0.001) (Fig. 8a–c). Iba1 is considered a constitutive marker for microglia, which is highly increased in 5xFAD animals compared to WT. [55, 56] Furthermore, it has been shown to be important for membrane ruffling, a process crucial for macrophage and microglia motility and chemotaxis [57]. We further characterized microglia based on ramifications and sphericity. The analysis showed that microglia of AAV-CERT_L_-injected mice had longer ramifications and lower sphericity index (Fig. 8d, e). To investigate if AAV-CERT_L_ affected other microglia membrane markers, we analyzed cortex tissue by RT-PCR. We quantified the CD86 membrane marker, which is an indicator for microglia pro-inflammatory polarization. Statistical analysis showed that CD86 was increased in AAV-control-treated 5xFAD animals compared to AAV-control-treated WTs and that AAV-CERT_L_ specifically decreased CD86 in the 5xFAD group (Fig. 8f).

Next, we analyzed cytokines levels in brain homogenate using multiplex technology. The assay did not reveal any significant effect of AAV-CERT_L_ treatment. However, we observed a significant increment of IL-1β (*p* < 0.01) and a significant reduction of IL-4 (*p* < 0.05) when comparing WT to 5xFAD animals in AAV-control treated groups (Supplementary Figure 6A).

These results indicate overall that AAV-CERT_L_ decreases pro-inflammatory processes in microglia.

### AAV-CERT_L_ does not change the immunoreactivity of astrocytes as shown by GFAP labeling in the cortex of 5xFAD mice

Reactive astrocytes have been described as a pathological hallmark that generally occurs in response to neurodegeneration in AD [58]. To determine the extent of astrocytosis in the 5xFAD mouse brain treated with AAV-CERT_L_ compared to control, we performed immunofluorescence GFAP labeling on sagittal brain sections (Fig. 8g). Densitometric and particle analysis performed with ImageJ showed a 47% reduction in astrocyte immunoreactivity, which was not statistically significant (Fig. 6h). However, as previously reported, we observed a 3-fold increase of GFAP immunoreactivity in the 5xFAD animals compared to WT controls [32, 59].

## Discussion

In this work, we provide evidence suggesting that CERT_L_ plays an important role in characteristic processes of AD by affecting Aβ production and aggregation, neuroinflammation, and SL disbalance typical of AD. Our data show that the pathological increase of Cer d18:1/16:0 can be reduced to normal levels by upregulating CERT_L_. The reduction of Cer levels also proved to be effective in attenuating Aβ formation. Furthermore, CERT_L_ overexpression in neurons revealed that CERT_L_ can downregulate membrane markers indicating a pro-inflammatory status in microglia.

It has previously been reported that APP can interact with extracellular matrix proteins like collagen I [60, 61]. This interaction is thought to be important in neuronal cell to cell adhesion with APP functioning as an anchor [62]. CERT_L_ is known to be crucial in stabilizing the basal membrane [63]. We previously reported that CERTs can be found in close location to plaques in AD brains, where they co-localize with amyloid fibrils [29]. In line with this, we found that APP can interact with CERT_L_ and form complexes that can be immune-precipitated. Also Aβ can bind to a variety of biomolecules, including lipids, proteins, and proteoglycans [64]. Here, we show that CERT_L_ binds directly to Aβ peptides and that this interaction affects Aβ fibrillization by organizing Aβ into less neurotoxic aggregates.

Manipulation of CNS SL metabolism can be challenging. Removal or addition of genes encoding for enzymes and transporters in the SL pathway can be deleterious for brain function [65, 66]. A previous report where human acid sphingomyelinase was increased in the brain of rodents and primates showed that motor function could be severely affected [67]. In contrast, in our study, behavioral testing at 9–10 weeks post AAV particle injection revealed no detectable side effects when comparing AAV-CERT_L_- to AAV-control-treated animals in various memory- and anxiety-related behavioral tasks. Of note though, we found a significant reduction of locomotor activity in the 5xFAD due to AAV-CERT_L_ treatment. It has been reported that 5xFAD animals exhibit a hyperactive behavior compared to control animals even though this specific behavior is not well understood and translation to human symptoms is unclear [68]. However, the question whether AAV-CERT_L_ could improve memory or other behavioral deficits observed in the 5xFAD model could not be answered by our data. No significant impairments in spatial memory were detected in the AYM and the SYM tests. In the original 5xFAD line (on a hybrid B6SJL background and carrying the Pde6b gene), Jawhar et al. reported deficits of working memory, assessed by a cross-maze test, to appear at 6 months [69]. In contrast, the behavioral phenotype of the 5xFAD line on a C57BL/6J background and without the Pde6b gene, are much less clear. Richard et al. reported in this last line of 5xFAD spatial memory deficits were only visible after 7 months of age [70]. Others reported spatial memory impairment at an earlier age when using the water maze but with a very small difference between WT and 5xFAD [71, 72]. In the present study, none of the groups tested in the SYM test performed above chance level indicating lack of recognition in all groups examined. However, in the AYM, the WT group treated with AAV-control performed above chance level (mean of 68%), which in the AYM is considered to be 50%. As such, future studies should additionally assess shorter inter-trial intervals in this respect.

Cer generation is abnormal in AD, causing an increase in Cer formation [15, 73]. Furthermore, an accumulating body of evidence consistently reported a global rise in Cer levels in specific brain regions of AD patients [13, 74, 75]. In agreement with these observations, we found that 5xFAD transgenic mice at 6 months of age also showed an increase of Cer d18:1/16:0 in the cortex and of several Cer species in the hippocampus (Cer d18:1/16:0, Cer d18:1/18:1, Cer d18:1/20:0 and Cer d18:1/22:0). Previously, other studies showed increased brain Cer levels in different transgenic AD models (APP, PS1, and PS1-APP mutated mice) [76]. This suggests that there is a causal relationship between amyloid pathology and Cer imbalance.

In this study, we report that increasing Cer trafficking from the ER to the Golgi by overexpressing CERT_L_ reversed the pathological increase of Cer in the cortex. A similar effect of CERTs overexpression in Cer elevation state has been reported in a lipotoxic mouse model where muscle cells in overload Cer status were rescued by increasing the expression of hCERT [52]. In AD brains, we observed that CERT_L_ reduced 11% of the total Cer content restoring it close to normal levels. In particular, Cer d18:1/16:0 was the most affected species by AAV-CERT_L_ being reduced up to 35%. These results demonstrate the importance of physiological ER-to-Golgi Cer traffic in preserving the physiological balance of Cer levels in AD pathology.

The soluble Aβ forms were reduced in the CERT_L_-treated animals, whereas the insoluble Aβ forms were not altered by the treatment. This reduction in the soluble forms could be explained in two ways: (i) the 11% reduction of total Cer due to CERT_L_ overexpression and (ii) CERT_L_ interaction with Aβ. As aforementioned, the amyloidogenic cleavage of APP is favored resulting in more Aβ formation in Cer enriched conditions [21, 22]. In our study, the total Cer reduction may have affected the secretases activity in the opposite way. We found that the Aβ/APP ratio, which describes the APP processing to form Aβ, was lower in AAV-CERT_L_ animals implying that lesser APP is processed to generate the Aβ peptide. Importantly, APP processing takes place in different cell compartments not only on the cell surface [77] and the Cer shift is not confined to the ER compartment but can affect the whole cell [78, 79]. This conclusion was further confirmed by pharmacological inhibition of CERT Cer transfer activity with CERT inhibitor HPA-12. Recently, the pharmacokinetics of HPA-12 was described, and it was proven that the compound reaches the brain intact [25]. Here, we found that after 4 weeks, the treatment of HPA-12 increases Cer and Aβ levels.

It is now thought that one of the crucial processes in the development and exacerbation of AD is neuroinflammation [80]. Our lab demonstrated that CERT_L_ interacts with SAP which belongs to the pentraxin family of the innate immune system [29]. Additionally, we reported that CERT_L_ can activate the complement system [31]. Here, we found that AAV-CERT_L_ influenced microglia activation even though CERT_L_ was specifically expressed in neurons under the control of the synapsin promoter. It has been consistently reported that 5xFAD microglia are polarized towards a more pro-inflammatory status, in response to the extensive plaque formation. Consequently, the Iba1 microglia marker is highly expressed in AD models [55, 56]. Our findings suggest that CERT_L_ could play a role in the cross-talk between neurons and microglia. Interestingly, neuronal-derived CERT_L_ activity is exerted only when there is an inflammatory reaction ongoing by reducing membrane markers for the pro/inflammatory status of microglia. Nevertheless, it remains unclear by which mechanism AAV-CERT_L_ decreased Iba1- and CD86-positive cells. Here, we propose two hypotheses. First, the reduction of Iba1 and CD86 is a direct action of CERT_L_ on microglia activation status once secreted by neurons. It is known that forms of CERT_L_ can be released in the extracellular space [30]. The second, Iba1 and CD86 are decreased because of a modified by shifts in Cer and SM composition or other indirect effects like reduction of Aβ levels. In the CNS, there is an extensive cross-talk ongoing between neurons and microglia, which takes advantage of lipid vesicles. Furthermore, toxic Aβ has also been reported among the content of exosome and reduction of exosome secretion was correlated to Aβ reduction [81, 82].

Similarly, to microglia, also astrocyte activation was reduced by AAV-CERT_L_, even though not significantly. During inflammation astrocytes are enriched in Cer. They seem to produce the pro-apoptotic Cer d18:1/16:0 [83, 84]. Further, reactive astrocytes release extracellular vesicle enriched in Cer that carry Aβ peptides [84]. These specific extracellular vesicles isolated from brains of 5xFAD mice showed to be particularly toxic for neurons.

### Limitations

The limitations of this study are three folded. First, we did not detect memory difference between WT and 5xFAD. This could be due to our in-house breeding approach, which is explained in material and methods section. We adopted a breeding scheme where the Pde6b gene, which is associated to retinal degeneration, was bred out of the genetic background of the 5xFAD transgenic mice. The Pde6b gene is thought to be crucial for early detection of memory impairments. The second limitation is connected to the first. Since no memory impairment was detected, it is unclear if the AAV-CERT_L_ would protect from memory decline. For this purpose, animals could be tested at older age and the AAV-CERT_L_ could be injected in the hippocampus. This follow-up study would be of interest also because the hippocampus, as we show for the first time in this study, is particularly affected by the Cer pathology in 5xFAD males. The third limitation is the effect of AAV-CERT_L_ on astrocytes. While we had sufficient statistical power to detect the microglia changes, astrocytes denoted a similar trend, which was not statistically significant. These three limitations set the ground for future studies.

## Conclusion

**Fig. 9:**
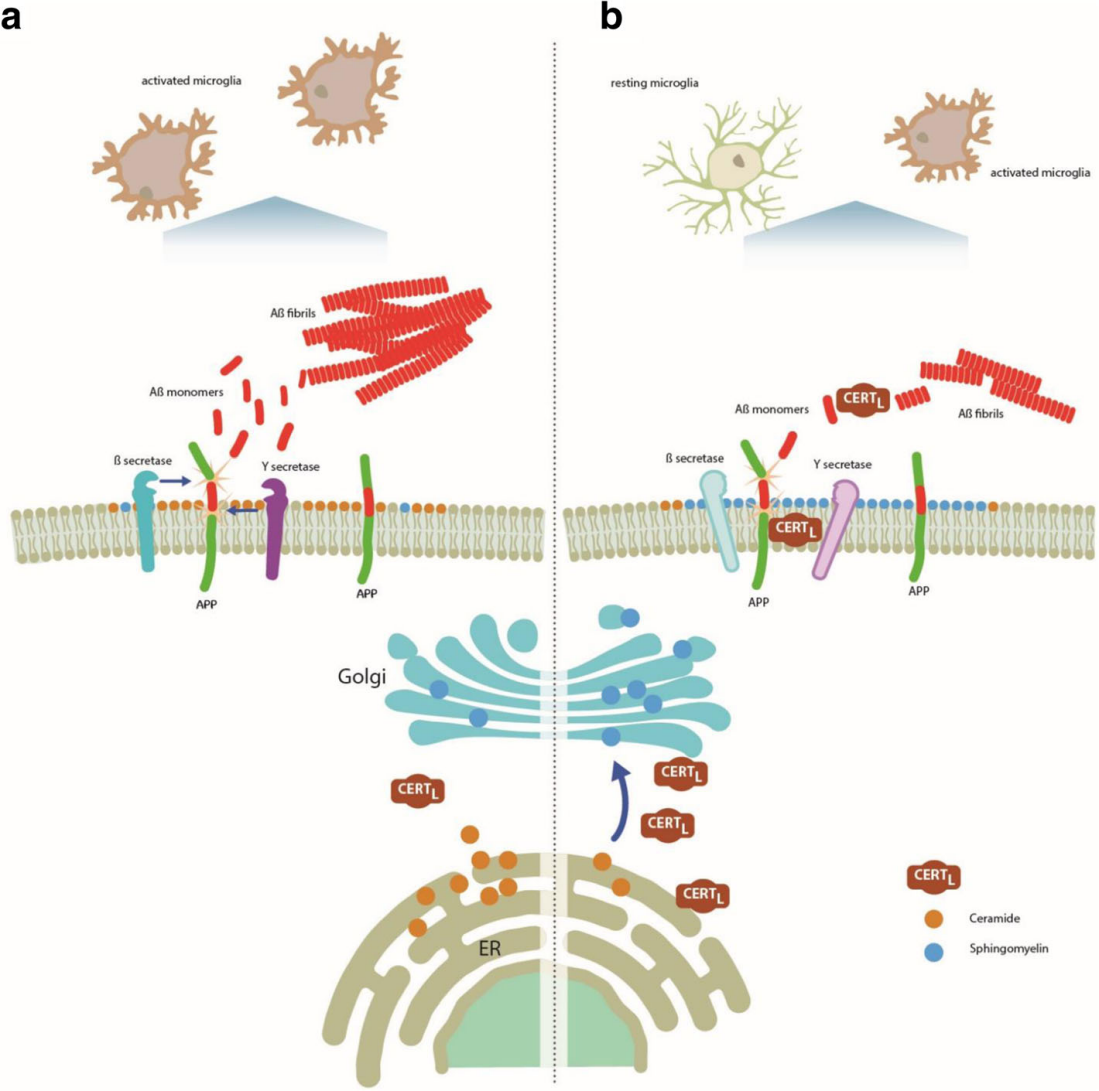
Schematic model of CERT_L_ action in AD. **a** CERT_L_ concentration is decreased in AD neuronal cells. Consequently, the transport of Cer to the Golgi is impaired and Cer accumulates in the cell. Cer elevation stabilizes and favors the secretases activity. The amyloidogenic APP processing is favored and Aβ is produced. The neighboring microglia changes the resting status to activate. **b** By overexpressing CERT_L_, the physiological transfer of Cer from the ER to the Golgi is restored favoring SM synthesis, which is intensified. The reduction of Cer levels in neuronal cells diminished secretases activity, reducing Aβ biogenesis. The interaction between CERT_L_ and APP may be important in stabilizing APP in the membrane and in protecting APP from secretase activity. Furthermore, CERT_L_ affects Aβ fibrilization by organizing Aβ into less neurotoxic aggregates that may be cleared from the brain more easily and reduces the number of activated microglia

In conclusion, by increasing CERT_L_ expression in neuronal cells, we were able to increase SM production and reduce Cer d18:1/16:0 especially in the CNS. Next, after proving that CERT_L_ binds and modifies Aβ aggregation in vitro, we observed that administration of AAV-CERT_L_ in AD animals reduced Aβ production by at least 2 mechanisms: by altering SL composition and by direct interaction with APP in 5xFAD animals. Moreover, we reported a new immune role of CERT_L_. AAV-CERT_L_ decreased membrane markers important for the pro/inflammatory status of microglia. Overall, our experiments are the first to demonstrate that an increase of CERT_L_ modulates SL levels and affects amyloid plaque formation and brain inflammation in AD (see the model in Fig. 9). These data open research pathways for therapeutic targets of AD and related neurodegenerative diseases.

## Supplementary Information

**Additional file 1: Supplementary methods**.

**Additional file 2: Supplementary Tables**.

**Additional file 3: Supplementary Figures**.

## Data Availability

The datasets used and/or analyzed during the current study are available from the corresponding author on reasonable request.

## Abbreviations

AD: Alzheimer’s disease
Aβ: Amyloid-β peptides
NFTs: Neurofibrillary tangles
BBB: Blood-brain barrier
SLs: Sphingolipids
Cer: Ceramide
S1P: Sphingosine-1-phosphate
SM: Sphingomyelin
CERTs: Ceramide transfer proteins
CERT_L_: Ceramide transfer proteins long form
START: Steroidogenic acute regulatory protein (StAR)-related lipid transfer
ER: Endoplasmic reticulum
AAV: Adeno-associated virus
IP: Immunoprecipitation
APP: Amyloid precursor protein
FL-APP: Full length amyloid precursor protein
CTFβ: C-terminal fragment-β
MST: Microscale thermophoresis
TEM: Transmission electron microscopy
ThT: Thioflavin T
OF: Open field
AYM: Y-maze spontaneous alternation
EZM: Elevated zero-maze
SYM: Y-maze spatial memory test
HPLC-MS/MS: High pressure liquid chromatography-tandem mass spectrometry
(mAbs): Monoclonal antibodies
(HPA-12): N-(3-Hydroxy-1-hydroxymethyl-3-phenylpropyl) dodecanamide
