# Supplementary material for: CERTL reduces C16 ceramide, amyloid-β levels, and inflammation in a model of Alzheimer’s disease
Source: Alzheimers Res Ther. 2021 Feb 17;13:45. doi: 10.1186/s13195-021-00780-0 (PMC7890977; doi:10.1186/s13195-021-00780-0)
Supplement: Supplementary file 1 — Additional file 1: Supplementary methods. [file 13195_2021_780_MOESM1_ESM.docx]

# Supplementary methods

*Co-localization of CERTs and APP/Aβ in 5xFAD brain*

Immunofluorescent staining was performed in 10 µm thin sagittal brain section of 3 months old males 5xFAD. The sections were fixed with acetone 10 minutes, blocked with 3% BSA for 1h and incubated overnight at 4 ^o^C with rabbit polyclonal anti-CERTS (epitope 300-350, Bethyl Laboratories) diluted 1:500 and anti APP/Aβ (clone 6E10 Covance) diluted 1:500. Next day sections were washed 3 times and the corresponding secondary antibodies conjugated to Cy2 or Cy3 (1:500) were applied for 1h at RT. After washing, the NeuroTrace 640/660 (ThermoFisher) was used following manufacturer instruction. Hoechst staining was performed for nuclei visualization. The slices were mounted with 80% glycerol and imaged.

*Lipidomics in CERT_L_ transfected neuroblastoma cells*

Neuro-2a (N2a) obtained from ATCC (CCL-131™)were cultured in DMEM supplemented with fetal bovine serum (FBS), Pen/Strep, and L-glutamine. Cells were seeded in 75 cm2 flasks and maintained in complete DMEM for 24 hours prior to the transfection. Cells were transfected with 2 µg pcDNA3.1-CERT_L_ (NM_005713.3) by Effectene Reagent (Qiagen) following the manufacturer’s instructions. Cells transfected with 2 µg of plasmid expressing GFP were used as control. After 48 hours, cells were washed three times with PBS, trypsinized, and centrifuged at 300 x g for 5 min. Pellet was stored at -80 before the lipid analysis. Sphingolipid analysis was performed as explained in Material and Methods section.

Far Western blot

For Far Western experiments, human recombinant CERT_L_ (hCERT_L_, 1875bp NP_005704.1) (80ng), produced as previously described ([29](#_ENREF_29)), and Aβ peptide 1-42 (80ng) (Anaspec) were analyzed by SDSPAGE under reducing conditions. A 17kDa Lama antibody fragment (H6) with myc and hist6 tags (a kind gift of Dr A.J. Groot), was used as a negative control (80ng). Proteins were transferred to nitrocellulose membranes renatured in Tris-buffered saline (TBS) in the presence of Tween20 (0.05%) and probed for 1 hour at 37°C with 30µg/mL of CERT_L_ or Aβ peptide in the same buffer. Bound complexes were detected using polyclonal rabbit anti-CERTs (epitope 1-50 of human CERTs, Bethyl Laboratories), mouse anti-Aβ mAb 6E10 (Covance) and mouse mAb anti-human c-Myc (clone 9E10, Invitrogen). Finally, incubation with secondary goat anti-rabbit IRdye 800 and donkey anti-mouse IRdye 680 (Rockland Immunochemicals) was followed as described above.

*Neuronal primary cell culture*.

Rat hippocampal neuronal cultures were prepared as reported elsewhere ([86](#_ENREF_86)). In brief, embryos from pregnant Wistar rats (Charles River) were removed on embryonic day 18. After removing the meninges, the hippocampi were insolated under the lupa preserved in the hibernate medium. Subsequently, they were incubated with trypsin for 20 minutes at 37 ºC. Hippocampus were then transferred to a tube with DMEM complete medium. With a sterile glass pipette, the cells were disbanded doing up and down until the tissue was disaggregated/disintegrated and homogeneous. Cells were plated at ~80,000 cell/ 35mm Ø plate containing coverslips and incubated in Neurobasal medium (supplemented with B27) (Thermo Fisher Scientific) for 2-3 weeks. The medium was partially exchanged 24 hours after and then once a week.

*Immunoassay for CERTs detection.*

CERTs were quantified by enzyme-linked immunoassay or Western blot. In brief, microplates were coated with 100 μL of polyclonal rabbit 01 (2,46 mg/mL) diluted 1:1000 using coating buffer (50 mM carbonate pH 9.6), sealed with a plastic sticker and incubated for 1 h at 37ºC. To build up the standard curve serial dilutions of recombinant CERTs, produced as previously explained ([29](#_ENREF_29)), were diluted in 10% BSA, 0.02% Tween-20 in PBS. Biotinylated polyclonal rabbit anti-CERTs 02, was used as a detection antibody diluted 1:500 in 1% BSA and 0.02% Tween-20 in PBS and incubated for 1 h at 37 ºC. Blocking, washes, and absorbance were performed as described in method sections immunoassays. Cortex sample were lysed in composed by 0.1% SDS, 0.1% Triton X-100, 1% glycerol, 1 mM EDTA, 1 mM EGTA, PhosSTOP and protein inhibitors (Roche). 40µg of protein extracted separated in SDS-PAGE electrophoresis and blotted onto nitrocellulose membrane. The membrane was probed with anti-GAPDH antibody (10R-G109A, Fitzgerald) as internal control and anti CERTs (epitope 300-350 of human CERTs, Bethyl Laboratories). After incubation with donkey anti-mouse, IRdye680, and anti-rabbit IRdye800 (Rockland Immunochemicals) diluted 1:10,000 in Odyssey blocking buffer, the membrane was scanned and analyzed with Odyssey imager Li-Cor. The intensities were measured with Odyssey imager Li-Cor.

CERTs inhibitor administration

HPA-12 was prepared in control solution PEG400/PBS 1:4 (V/V). Before re-suspension vehicle was sterilized by filtration and pH was adjusted to 7.4. PEG400 / PBS was warmed up to 37°C and vortexed to completely dissolve the compound. HPA-12 was administered subcutaneously (SC) for 4-weeks at the dose 4 µg / g animals every 48 hours. The volume injected per animal was of 0.15 mL with insulin syringes. HPA-12 was tested on 8-14 months old mice. The AD transgenic (E4FAD and E3FAD) mice were purchased from Dr. Mary Jo LaDu (University of Illinois at Chicago) and bred in house as described elsewhere ([87](#_ENREF_87)). The mice were housed socially on a 12-hour day-night cycle and had *ad libitum* access to food and water. All experiments were approved by the Animal Welfare Committee of Maastricht University and followed the laws, rules, and guidelines of the Netherlands.

## *Cytokines analysis*

Cytokines were measured in total protein homogenate extracted from the cortex in lysis buffer containing 0.1% SDS, 0.1% Triton X-100, 1% glycerol, 1 mM EDTA, 1 mM EGTA, phosphatase and protease inhibitors. Samples were diluted to 0.1mg / mL total protein and cytokines (IFN-γ, IL-1β, TNF-α, IL-6, IL-4, IL-10, and IL-33) were measured on Meso Scale Discovery V-PLEX custom mice (K152A0H-1) and read on a Meso Scale Discovery SECTOR 600 (Meso Scale Discovery).
