# Supplementary material for: CERTL reduces C16 ceramide, amyloid-β levels, and inflammation in a model of Alzheimer’s disease
Source: Alzheimers Res Ther. 2021 Feb 17;13:45. doi: 10.1186/s13195-021-00780-0 (PMC7890977; doi:10.1186/s13195-021-00780-0)
Supplement: Supplementary file 2 — Additional file 2: Supplementary Tables. [file 13195_2021_780_MOESM2_ESM.docx]

| **Cortex** | | | | | **Hippocampus** | | | | | **Cerebellum** | | | | |  |
| --- | --- | --- | --- | --- | --- | --- | --- | --- | --- | --- | --- | --- | --- | --- | --- |
| **WT (N=11)** | | **5xFAD (N=12)** | |  | **WT (N=11)** | | **5xFAD (N=12)** | |  | **WT (N=11)** | | **5xFAD (N=12)** | |  |  |
| **Sphingolipid species** | **Mean** | **STDEV** | **Mean** | **STDEV** | **p-value** | **Mean** | **STDEV** | **Mean** | **STDEV** | **p-value** | **Mean** | **STDEV** | **Mean** | **STDEV** | **p-value** |
| **SPH** | 0.3 | 0.2 | 0.3 | 0.1 | *0.9475* | 0.2 | 0.1 | 0.3 | 0.1 | *0.2347* | 0.4 | 0.1 | 0.3 | 0.1 | *0.1823* |
| **SPA** | 0.5 | 0.2 | 0.5 | 0.2 | *0.5042* | 0.3 | 0.1 | 0.5 | 0.2 | **↑0.0216*** | 0.4 | 0.2 | 0.4 | 0.1 | *0.9206* |
| **S1P** | 2.8 | 1.6 | 3.0 | 1.6 | *0.8084* | 2.0 | 0.8 | 3.0 | 1.8 | **↑****0.0038*** | 3.2 | 1.5 | 3.6 | 2.3 | **↑0.0123*** |
| **Cer d18:1/14:0** | 0.1 | 0.1 | 0.1 | 0.0 | *0.8960* | 0.0 | 0.0 | 0.0 | 0.0 | *0.4043* | 0.0 | 0.0 | 0.0 | 0.0 | *0.5884* |
| **Cer d18:1/16:0** | 0.8 | 0.3 | 1.1 | 0.3 | **↑0.0080*** | 0.6 | 0.3 | 0.8 | 0.2 | **↑0.0400*** | 0.5 | 0.3 | 0.6 | 0.2 | *0.6812* |
| **Cer d18:1/18:1** | 0.2 | 0.1 | 0.2 | 0.1 | *0.0531* | 0.1 | 0.1 | 0.2 | 0.1 | **↑0.0050*** | 0.1 | 0.0 | 0.1 | 0.0 | *0.9309* |
| **Cer d18:1/18:0** | 40.1 | 14.0 | 43.9 | 10.5 | *0.4833* | 36.8 | 10.8 | 43.7 | 15.8 | *0.0959* | 14.6 | 4.7 | 14.3 | 3.9 | *0.8843* |
| **Cer d18:1/20:0** | 1.4 | 0.4 | 1.7 | 0.4 | *0.1361* | 1.0 | 0.3 | 1.4 | 0.3 | **↑0.0041*** | 0.8 | 0.2 | 0.9 | 0.2 | *0.6595* |
| **Cer d18:1/22:0** | 0.6 | 0.2 | 0.7 | 0.2 | *0.1888* | 0.4 | 0.2 | 0.6 | 0.2 | **↑0.0222*** | 0.6 | 0.2 | 0.6 | 0.2 | *0.9508* |
| **Cer d18:1/24:1** | 50.5 | 25.0 | 55.9 | 20.6 | *0.5752* | 31.3 | 11.1 | 32.1 | 9.6 | *0.7817* | 101.7 | 57.9 | 90.7 | 36.9 | *0.5888* |
| **Cer d18:1/24:0** | 0.4 | 0.2 | 0.5 | 0.2 | *0.5214* | 0.2 | 0.1 | 0.4 | 0.2 | *0.0666* | 0.5 | 0.2 | 0.5 | 0.2 | *0.9824* |
| **Total SM** | 2592.1 | 1221.9 | 2895.1 | 888.2 | *0.2631* | 4041.1 | 1926.9 | 5050.3 | 3730.6 | *0.4308* | - | - | - | - | - |

**Supplementary Table 1** Sphingolipid levels on cortex, hippocampus, and cerebellum of WT and 5xFAD treated with AAV-control

(student′s t‐test*p<0.05)

↑ 5xFAD mean significantly bigger than WT animals

| Gene | GenBank Accession No. | Sequence (5'->3') |  |
| --- | --- | --- | --- |
| Bax | [XM_011250780.2](https://www.ncbi.nlm.nih.gov/entrez/viewer.fcgi?db=nucleotide&id=1039777052) | CACCTGAGCTGACCTTGGAG | Forward |
|  |  | CCACGTCAGCAATCATCCTCT | Reverse |
| Caspase 3 | NM_009810 | TGCAGCATGCTAAGCTGTA | Forward |
|  |  | GAGCATGGACAATACACG | Reverse |
| Bcl-2 | NM_009741 | TGGGATGCCTTTGTGGAACT | Forward |
|  |  | GAGACAGCCAGGAGAAATCA | Reverse |
| CERT_L_ | [NM_023420.2](https://www.ncbi.nlm.nih.gov/entrez/viewer.fcgi?db=nucleotide&id=255982522) | ATGTTCACAGATTCAGCTCCC | Forward |
|  |  | CTTCTTCAACAACCAGTTGCC | Reverse |
| CERT | [XM_011244694.2](https://www.ncbi.nlm.nih.gov/entrez/viewer.fcgi?db=nucleotide&id=1039744052) | CAGGATGTAGGTGGTGATGC | Forward |
|  |  | CACCTTTAACTGCATGAGTAGC | Reverse |
| GAPDH | XM_017321385.1 | CTCATGACCACAGTCCATGC | Forward |
|  |  | TTCAGCTCTGGGATGACCTT | Reverse |
| Actin | [NM_007393.5](https://www.ncbi.nlm.nih.gov/entrez/viewer.fcgi?db=nucleotide&id=930945786) | CTCTCAGCTGTGGTGGTGAA | Forward |
|  |  | AGCCATGTACGTAGCCATCC | Reverse |
| CD86 | NM_019388.3 | GGGCTTGGCAATCCTTATCT | Forward |
|  |  | ACCAACTTTTGCTGGTCCTG | Reverse |
| Fizz-1 | NM_020509.3 | GGAACTTCTTGCCAATCCAG | Forward |
|  |  | ACACCCAGTAGCAGTCATCCC | Reverse |

**Supplementary Table 2. RT-PCR primer sequences**
