# Supplementary material for: CERTL reduces C16 ceramide, amyloid-β levels, and inflammation in a model of Alzheimer’s disease
Source: Alzheimers Res Ther. 2021 Feb 17;13:45. doi: 10.1186/s13195-021-00780-0 (PMC7890977; doi:10.1186/s13195-021-00780-0)
Supplement: Supplementary file 3 — Additional file 3: Supplementary Figures. [file 13195_2021_780_MOESM3_ESM.docx]

# Supplementary figures


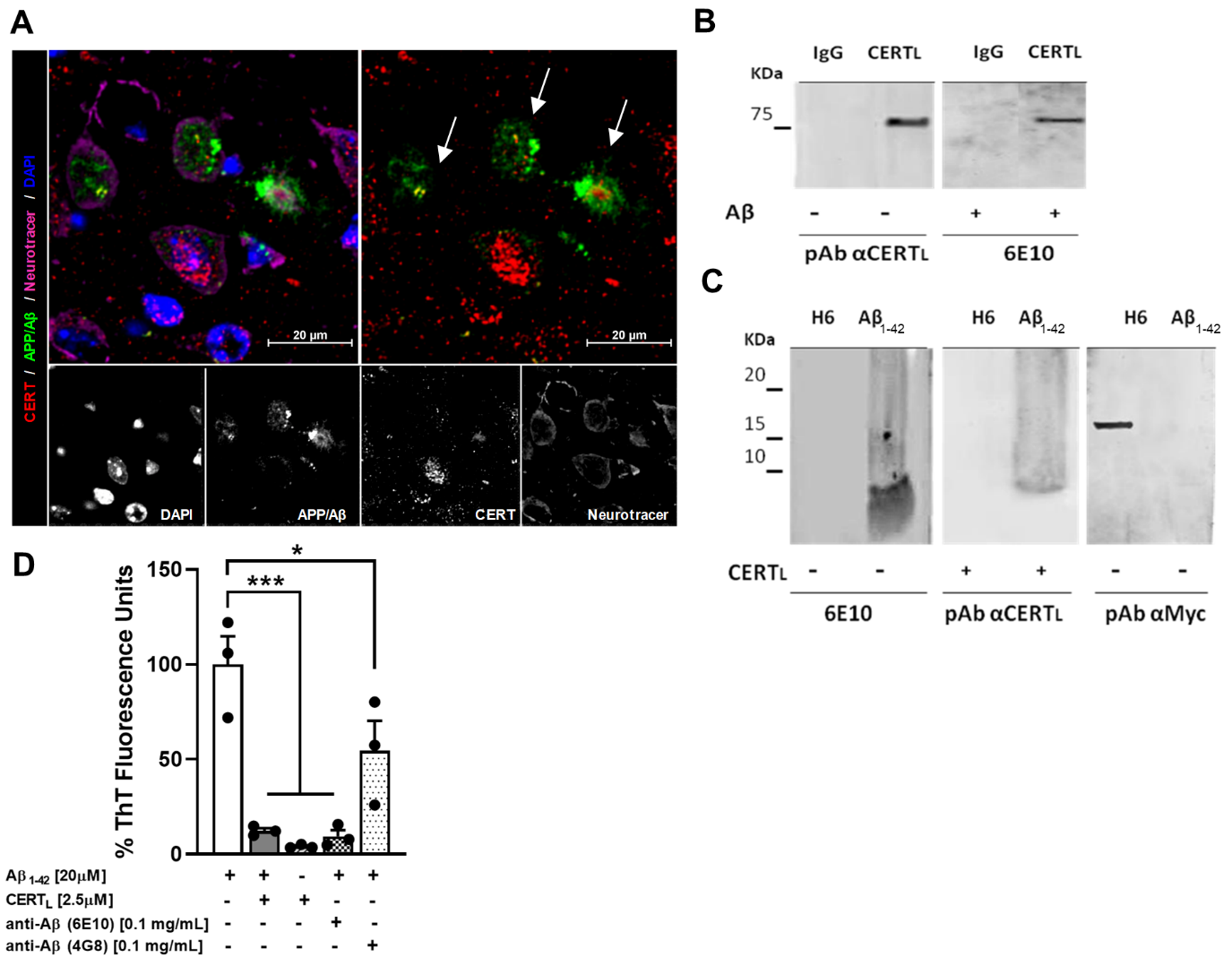


**Supplementary figure 1. Immunostaining of CERT_L_ in the brain and analysis of CERT_L_ Aβ interaction by far Western blot.** A) Photomicrographs of a 5xFAD brain section showing CERT and APP/Aβ colocalization intra and extra neurons. CERT is shown in red, APP/Aβ in green, neurons in magenta and nuclei in blue. Arrows point to intra and extra-neuronal colocalization B) Image showing detection of recombinant CERT_L_ separated by SDS-PAGE, transferred to membrane and detected with anti-CERT_L_ antibody without Aβ_1-42_ peptide pre-incubation. CERT_L_ was detected with anti-Aβ antibody after Aβ_1-42_ peptide pre-incubation. IgG remained negative control. C) Image showing Aβ_1-42_ peptide separated by SDS-PAGE, transferred to membrane and detected with anti-Aβ antibody (6E10). After recombinant CERT_L_ incubation, Aβ_1-42_ peptide was immunolabeled with anti-CERT_L_ antibody but not with a 17 kDa Lama antibody fragment (H6) (negative control). D) Percentage of Thioflavin T (ThT) fluorescence intensity to detect aggregation of Aβ_1-42_, concentrated 20 µM, alone or in presence of recombinant CERT_L_ 2.5 µM, anti- Aβ epitope 1-16 (6E10) or 17-24 (4G8), dosed 0.1mg/mL, at 20 hours. Each data point represents the percentage of mean fluorescent intensity of three wells. (ANOVA; Dunnett's multiple comparisons test *p< 0.05, ***p<0.001)


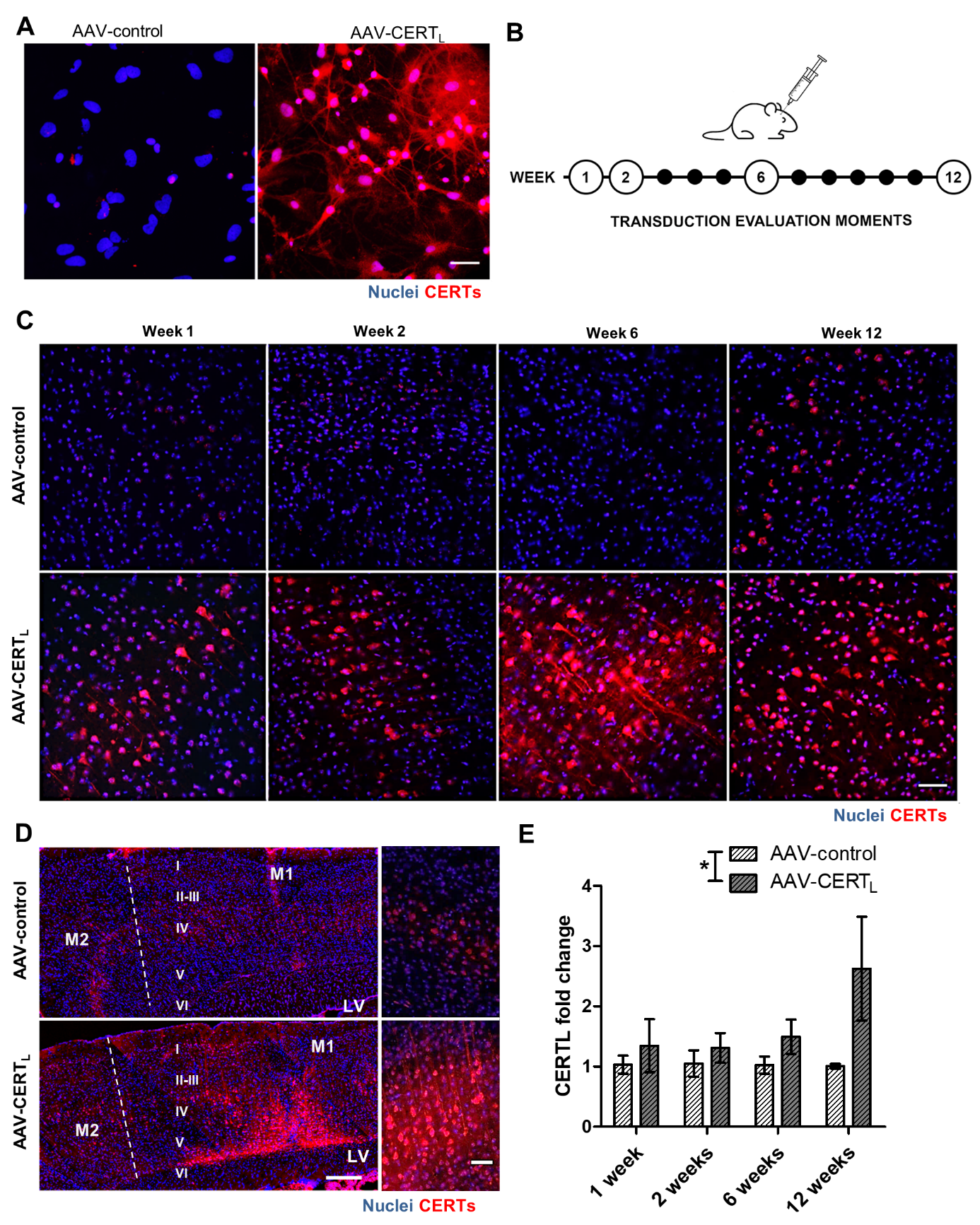


**Supplementary figure 2. CERTs concentration in WT compared to 5xFAD and evidence of CERT_L_ transduction *in vitro* and *in vivo*.** A) AAVs transduction on neuronal primary cell culture. Neonatal rat neuronal cells were isolated from the cortex of pups at embryonic day 18. After 2-3 weeks in Neurobasal medium were incubated with AAVs particles at the dose of 100 multiplicity of infection (MOI) for 6 days. CERT_L_ was visualized in red and nuclei in blue. B) A timeline describing the experimental design to assess transduction efficiency *in vivo*. C) AAVs transduction in WT animals 1, 2, 6- and 12-weeks post-injection by immunofluorescence. CERT_s_ were visualized in red and nuclei in blue (scale bar 50 μm). D) Immunofluorescence staining on brain sections showing injections site (CERT**_L_** in red and nuclei in blue). Scale bars 200 and 50 μm. **M1**: motor sensory cortex 1; **M2**: motor sensory cortex 2; **LV**: lateral ventricle; **I, II, III, IV,** and **V** cortical layers. E) AAVs were tested for brain transduction in WT animals 1, 2, 6- and 12-weeks post-injection by qPCR. Bars represent the mean ± S.E.M 3/per group. A significant increase in CERT_L_ transduction was measured independently from time point (ANOVA; *p< 0.05).


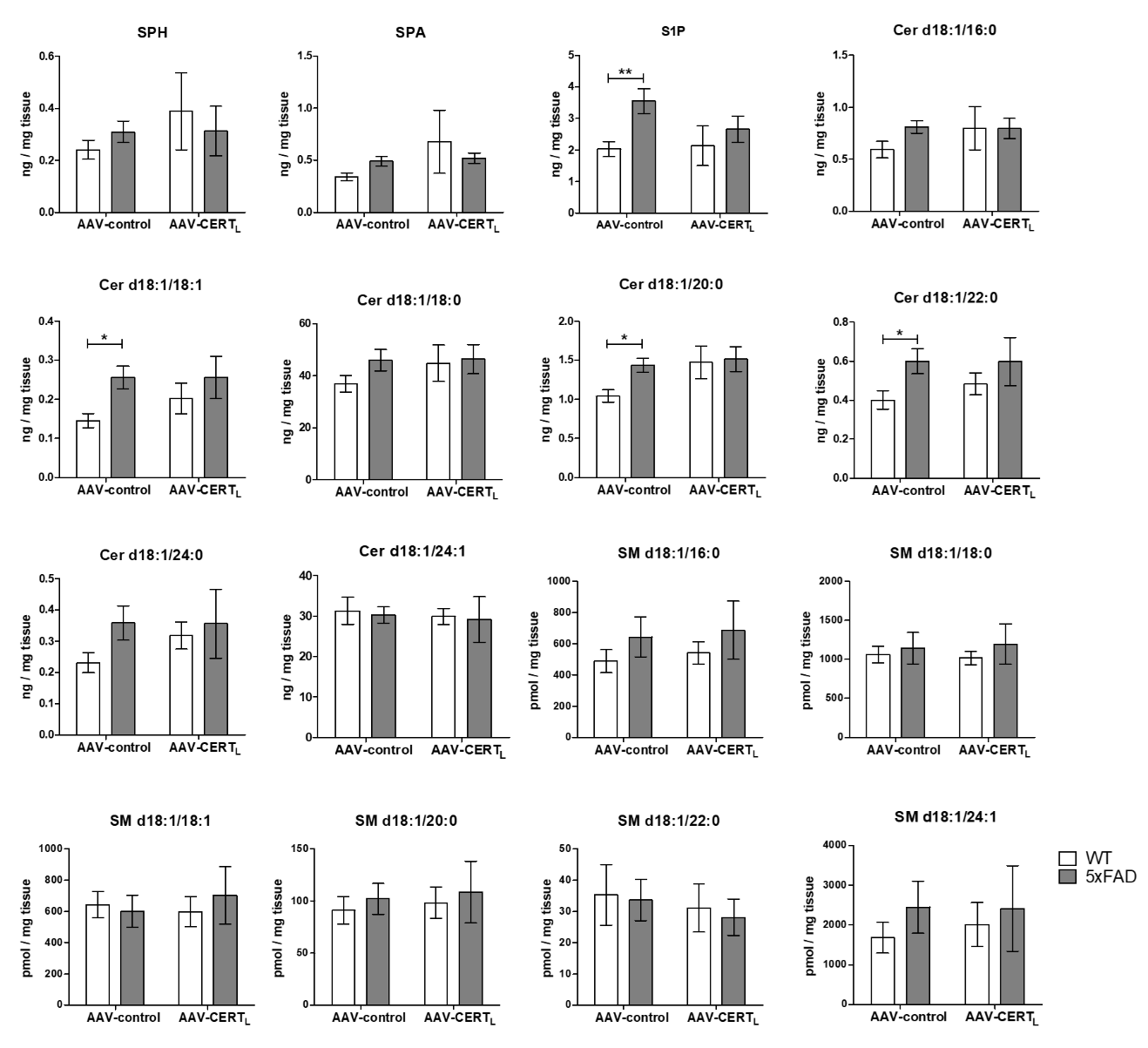


**Supplementary figure 3. Sphingolipid changes in the hippocampus region of AAV-control or AAV-CERT_L_ treated WT and 5xFAD mice.** Sphingolipids levels were measured in the hippocampus by HPLC-MS/MS. Ceramides were classified based on acyl chain number of carbons (Cer d18:1/16:0, Cer d18:1/18:0, Cer d18:1/18:1, Cer d18:1/20:0, Cer d18:1/22:0 and Cer d18:1/24:1). as well as sphingomyelin (SM d18:1/16:0, SM d18:1/18:0, SM d18:1/18:1, SM d18:1/20:0, SM d18:1/22:0 and SM d18:1/24:1). Ceramides levels were expressed as pg / mg tissue, while sphingomyelins were expressed as pmol/mg tissue. Bars represent the mean ± S.E.M per group (Two-way ANOVA, LSD, significant effects, *p< 0.05; **p<0.01; ***p<0.001).


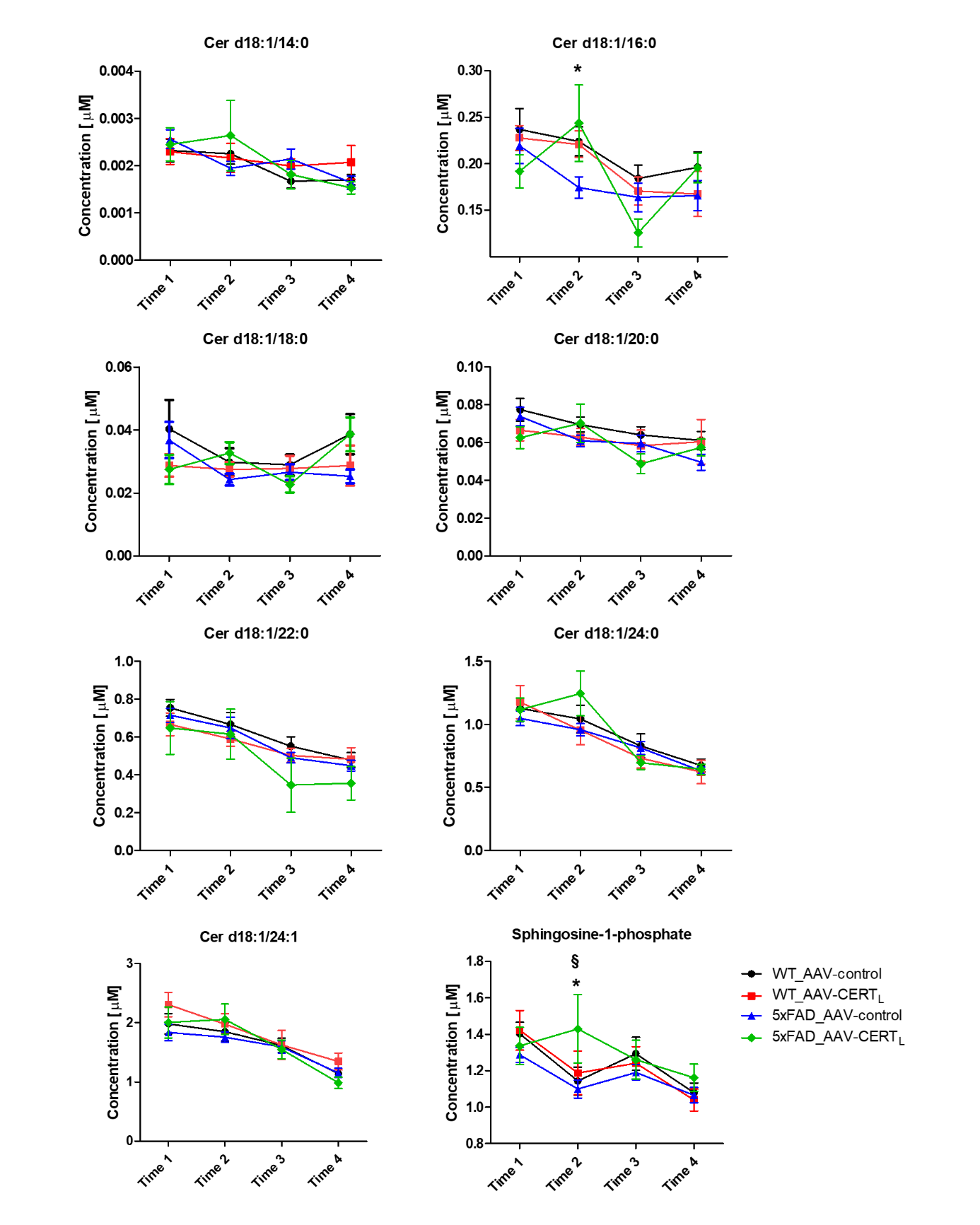


**Supplementary figure 4. Sphingolipid changes in plasma of AAV-control or AAV-CERT_L_ treated WT and 5xFAD mice.** Plasma was collected 1 week before AAV injection (time1), 2 (time 2), and 6 (time 3) weeks after injection, and when the experiment was ended (time 4). Sphingolipids levels were measured at each time point by HPLC-MS/MS. Ceramides were classified based on acyl chain number of carbons (Cer d18:1/16:0, Cer d18:1/18:0, Cer d18:1/18:1, Cer d18:1/20:0, Cer d18:1/22:0 and Cer d18:1/24:1). Ceramides concentration is expressed in µ Molarity. Bars represent the mean ± S.E.M per group (Two-way ANOVA, Bonferroni correction, significant effects, WT AAV-control vs 5xFAD AAV-CERT_L_ §p<0.05; 5xFAD AAV-control vs 5xFAD AAV-CERT_L_ *p<0.05).


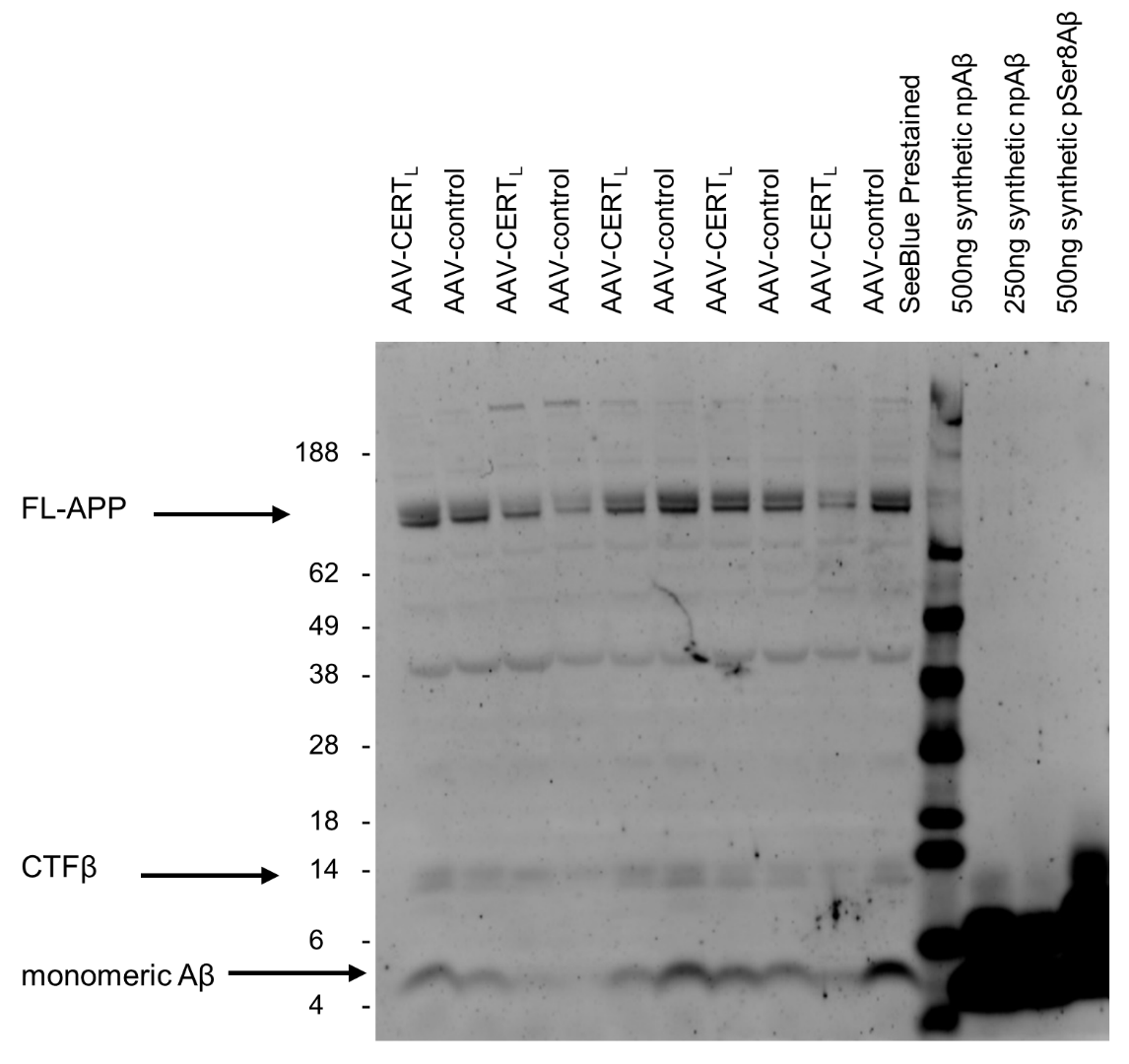


**Supplementary figure 5. Western blot analysis of APP fragments to study APP processing.** TBS cortex homogenate was analyzed by Western blot FL-APP, CTFβ, and Aβ bands were detected with 6E10 antibody at the band corresponding to respectively of 100, 15, and 6-4kDa.


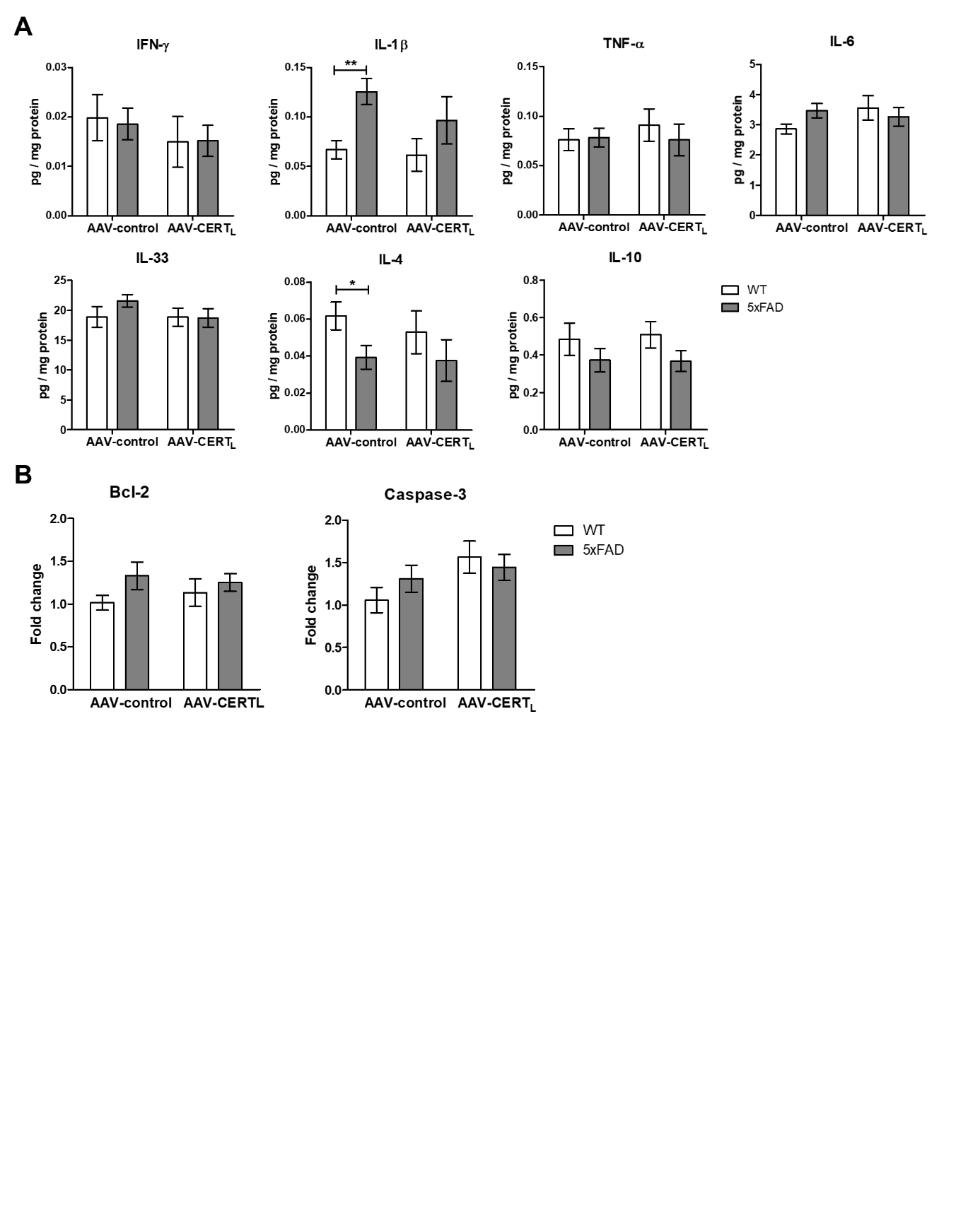


**Supplementary figure 6. Cytokines and apoptotic markers Bcl-2 and Caspase 3 were unaffected by AAV-CERT_L_ treatment.** A) Cytokines measurements in brain homogenate with multiplex system IFN-γ, IL-1β, TNF-α, IL-6, IL-33, IL-4, and IL-10 (5-10 number of animals per group). **B)** Analysis of gene expression of apoptotic markers Bcl-2 and Caspase-3 (4-5 number of animals per group, two-way ANOVA, Bonferroni correction).
